# Genomic Organization, Evolutionary Conservation and Expression of *Ataxin-2* and *Ataxin-2-like* Genes Underscore the Suitability of Zebrafish as a Model Organism for SCA2 and Related Diseases

**DOI:** 10.3390/biomedicines13122974

**Published:** 2025-12-03

**Authors:** Franz Vauti, Lukas Eilers, Anneke Kroll, Reinhard W. Köster

**Affiliations:** Cellular & Molecular Neurobiology, Zoological Institute, Technische Universität Braunschweig, Spielmannstraße 7, 38106 Braunschweig, Germany; eilers.lukas@mh-hannover.de (L.E.); anneke.kroll@tu-braunschweig.de (A.K.); r.koester@tu-braunschweig.de (R.W.K.)

**Keywords:** *atxn2*, *atxn2l*, zebrafish, spinocerebellar ataxia 2, SCA2, ALS, phylogenetic tree, gene structure, gene expression, whole-mount in situ hybridization, WISH, development

## Abstract

**Background/Objectives:** The Ataxin-2 protein (ATXN2) plays an essential role in RNA metabolism and many cellular processes. Dysregulation or mutation of the *Ataxin-2* gene (*ATXN2*) can lead to neurodegenerative diseases such as spinocerebellar ataxia type 2 (SCA2) and amyotrophic lateral sclerosis (ALS). Despite numerous efforts in this field in other animal models, little is known about Atxn2 in zebrafish. In this study, we aim to investigate the potential suitability of zebrafish as a model for Atxn2-related diseases by performing basic analyses on Atxn2. **Methods:** We performed a bioinformatic protein analysis of Atxn2 from zebrafish and its paralog Atxn2l in relation to human and other vertebrate homologues. Based on a structural analysis of the *atxn2* and *atxn2l* genes, the expression of the predicted transcripts was detected by RT-PCR and the spatiotemporal expression pattern was determined by whole-mount in situ hybridization. **Results:** We found similarities between the protein sequences of Atxn2 and Atxn2l in zebrafish and their functional domains with those of orthologs in humans and other vertebrates. The predicted transcripts of *atxn2* and *atxn2l* were experimentally verified and their spatiotemporal expression patterns were determined during zebrafish development. Splicing variants were detected for both genes, suggesting a different role for the isoforms in different tissues. **Conclusions:** These findings provide new insights into the *atxn2* and *atxn2l* genes, suggesting the zebrafish as a suitable animal model for functional studies and research on disease modeling of SCA2 and ALS.

## 1. Introduction

All eukaryotic organisms contain at least one copy of the *ataxin-2* (*ATXN2*) gene, whose encoded protein is known for its role in regulating mRNA stability [1,2,3]. During cellular stress, ATXN2 is transcriptionally induced and the protein is translocated from the endoplasmic reticulum to cytoplasmic stress granules [4,5]. The protein structure domains of ATXN2 have also been identified in another paralogue, ataxin-2-like (ATXN2L), a protein that plays a role in RNA surveillance in stress granules [6]. However, the physiological role of ATXN2L remains largely unclear.

The human *ATXN2* gene encodes a protein of 140 kDa [7,8,9]. The N-terminal region contains between 13 and 31 glutamine repeats, in most cases 22 consecutive glutamines (polyQ22), the length of which can be extended by mutations. Intermediate Q27–Q33 expansions in ATXN2 are associated with the risk of amyotrophic lateral sclerosis (ALS) and frontotemporal lobar dementia (FTLD) [10,11,12,13,14], with tauopathies and Parkinson’s variants such as progressive supranuclear palsy (PSP) [15,16]. Due to the genetic instability of a glutamine domain (polyQ) in ATXN2 across generations [17], an abnormally expanded polyQ in ATXN2 (>Q35) plays an important role in spinocerebellar ataxia type 2 (SCA2). SCA2 belongs to a complex group of late-onset neurodegenerative diseases in humans with loss of cellular homeostasis, which usually primarily affect the cerebellum [7,8,18], the brain stem, and the spinal cord [19,20]. Symptoms of SCA2 include slowly progressive abnormalities in motor coordination that affect limb and eye movements as well as speech.

The complete penetrance of SCA2 lies between 35 and 500 glutamine units in the polyQ segment [21]. Interestingly, the length of the polyglutamine segments correlates inversely with age at symptom onset and the occurrence of neuropathological signs [22,23]. The symptoms of SCA have been documented for at least 48 unrelated SCA disease loci to date [24]. Seven of these loci contain a CAG triplet expansion in their coding gene, resulting in an expanded polyQ in the translated gene products [25,26,27].

Like all forms of ataxia, SCA2 is a rare disease. The prevalence of SCA2 is estimated at one to two patients per 100,000 people, with variations depending on ethnicity and geographical region [28,29,30,31]. However, in some regions of the world, the rates may be significantly higher due to a founder effect [32,33]. A hotspot for SCA2 is the Holguin region in Cuba [34,35,36,37].

Despite numerous efforts in this field, there is no cure for SCA. Current knowledge of the molecular mechanisms underlying SCA2 comes largely from modeling SCA2 in animal models. *Atxn2^−/−^* knockout mice showed no histopathological defects of the central nervous system, and only minor deficits in motor behavior [38]. However, reduced fertility, motor hyperactivity, abdominal obesity, and hepatic steatosis were observed at six months of age [39]. Recent findings have shown that Atxn2 deficiency also affects the circadian system of mice [40] and flies [41,42].

Reduced expression of ATXN2 may also contribute to the pathogenesis of ALS. Mutated ATXN2 with specific polyQ expansions enhances the Transactive Response DNA-binding Protein 43 kDa (TDP-43) aggregation, thereby promoting ALS pathogenesis [43,44]. However, therapeutic administration of *Atxn2* antisense oligonucleotides or crossbreeding in *Atxn2^−/−^* knockout mice reduces TDP-43 aggregation, improves motor function, and prolongs the lifespan in animal models [10].

In recent years, the zebrafish *Danio rerio* has emerged as an attractive model for elucidating the function of vertebrate genes. Initial models related to SCA2 in zebrafish were described in knockdown studies of the *c9orf72* gene, which is involved in the pathogenesis of ALS-FTLD [45,46]. Recently CRISPR/Cas9-mediated knockdown studies in zebrafish showed that the loss of Atxn2 led to a reduction in eye size, a decrease in retinal ganglion cells, an increase in intraocular pressure, and impaired visual function, suggesting a possible new role for ATXN2 in the pathogenesis of primary open-angle glaucoma [47]. There is still a lack of knowledge about the *atxn2* and *atxn2l* genes in zebrafish. Although bony fish often have two copies of a gene due to genome duplication, this is not the case with *atxn2* and *atxn2l*, where only one gene is present. Based on this fact and our results, we propose the zebrafish as a suitable animal model for researching Atxn2- and Atxn2l-associated diseases.

## 2. Materials and Methods

### 2.1. Bioinformatics

Protein sequences of ATXN2 and ATXN2L protein sequences from humans, chimpanzees, rats, mice, chickens, frogs, zebrafish and killifish (Table S1) were downloaded from the Ensembl and NCBI database server. The phylogenetic analysis of the proteins was performed using the Unipro UGENE analysis tool using the PHYLIP Neighbour Joining method and the Jones-Taylor-Thornton distance matrix model [48]. Zebrafish proteins for Atxn2 (protein ID: ENSDARP00000078091.4) and Atxn2l (protein ID: ENSDARP00000115477.1) were also used for domain analysis using the CDD database (The Conserved Domain Database is a resource for the annotation of functional units in proteins) at NCBI [49]. The settings of the search parameters (database: CDSEARCH/oasis_pfam; limit value for the E-value: 0.01; composite-based customization: yes; filters with low complexity: yes) were selected only for concise results, domains with low complexity were omitted. Predicted protein domains and motifs were also identified using InterPro (https://www.ebi.ac.uk/interpro/; URL accessed on 25 August 2025) and the Pfam database [50,51]. Further analysis was performed on AlphaFold prediction models using UniProt Match A2CF31 (Atxn2) and UniProt Match F1QA42 (Atxn2l). AlphaFold generates a Per-Residual Confidence Score (pLDDT) between 0 and 100. The reliability of the model is indicated in colors. Dark blue: Very high (pLDDT > 90); light blue: confident (90 > pLDDT > 70); yellow: low (70 > pLDDT > 50) and orange: very low (pLDDT < 50). Some regions below 50 pLDDT may be unstructured in isolation. The genomic structures of the *atxn2* and *atxn2l* genes were obtained from the Ensembl database. The transcripts *atxn2* (*atxn 2* transcript ID: ENSDART00000083656.5) and *atxn2l* (*atxn 2-like* transcript ID: ENSDART00000133168.3) were downloaded from the Ensembl database server, and the predicted transcript sequences were retrieved from the NCBI database server. The transcript sequences were matched, and the consensus sequences were blasted using the NCBI (refseq_rna) database with reference to *Danio rerio* (taxid:7955).

### 2.2. Embryo and Larvae Treatment

For the matings, zebrafish males and females from the *brass* and *casper* lines were used. Fertilized eggs were collected in the fish water immediately after mating, and unfertilized eggs and cell debris were removed. Embryos and larvae were incubated in fish water in a humidified incubator at 28 °C. The fish water was replaced with 30% Danieau medium (about 10 h after fertilization) and 1-phenyl-2-thiurea (PTU) was added after gastrulation to suppress residual pigmentation in embryos of the *brass* line. Danieau/PTU medium was changed daily until embryos or larvae (up to 10 dpf) were used for experiments. The larvae were transferred from the incubator to the tanks in the fish room at 6–7 dpf. To avoid prolonged exposure of the *brass* line to PTU beyond 10 dpf of larval development, the transparent *casper* line was kept in the fish room in 30% Danieau medium without PTU until 17 dpf. Our zebrafish lines *brass* and *casper* have been maintained in accordance with local animal welfare standards (Tierschutzgesetz §11, Abs. 1, Nr. 1) and the animal welfare directives and legal regulations of the European Union (EU Directive 201_63). The zebrafish Danio rerio was kept under the supervision of the animal welfare office LAVES (Niedersächsisches Landesamt für Verbraucher-schutz und Lebensmittelsicherheit; Permit # AZ 32.5./325-1-5-6-1). Embryos and larvae were anesthetized with tricaine and killed in ice water according to protocols approved by the Niedersächsisches Landesamt für Verbraucherschutz und Lebensmittelsicherheit (Permit # Az: 33.19-42502-05-16A070).

### 2.3. RNA Isolation, RT-PCR, Cloning and Probe Synthesis

Using peqGOLD RNAPure™ (VWR, International GmbH (Avantor), Darmstadt, Germany), total RNA was isolated from tissue samples as follows: adult zebrafish, adult zebrafish brains, 1-cell zygotes, embryos at developmental stages of 3 hpf, 6 hpf, 12 hpf, 1 dpf, 2 dpf, 3 dpf, 4 dpf, and developmental larvae at stages of 5 dpf, 7 dpf, and 10 dpf. For reverse transcription of mRNA, 2 μg of total RNA was reverse transcribed using oligo(dT) primers, a dNTP mix and SuperScript™ III reverse transcriptase (Thermo Fisher Scientific, Darmstadt, Germany) and incubated for 1 h at 50 °C and 15 min at 55 °C. For PCR of *atxn2* and *atxn2l* transcripts, partial cDNA sequences were amplified with primers as indicated (Table S2). For the cloning of zebrafish constructs and the generation of riboprobes, RT-PCR products were isolated (QIAGEN Gel Extraction Kit, QIAGEN GmbH, Hilden, Deutschland) and the purified fragments were cloned into the pGEM-T^®^ Easy vector. Linerarized plasmids were transcribed with T7 or Sp6 RNA-polymerases and the DIG RNA Labelling Mix (Sigma-Aldrich Chemie GmbH, Taufkirchen, Germany) was used to generate DIG-labeled antisense and sense riboprobes. The riboprobes were cleaned with the RNeasy kit (QIAGEN GmbH, Hilden, Deutschland) and checked for quality after denaturing gel electrophoresis, as described elsewhere [52].

### 2.4. Whole Mount In Situ Hybridizations (WISH)

In situ hybridization was performed in embryos and larvae according to a recently published all-age whole-mount in situ hybridization protocol [52]. In summary, prior to pre-hybridization, embryos and larvae were stored in 100% methanol, rehydrated in decreasing rows of ethanol, permeabilized by acetone/xylene, and bleached in 6% H_2_O_2_. Denatured (80° C, 2 min) riboprobes (50 ng/mL) were added to the hybridization solution at 65° C for a longer period of 60 h. After washing, the tissues were incubated overnight in a dilution with anti-DIG antibodies (1:4000). Intensive washing steps were carried out for three days. BM purple (Roche) was used as a substrate for dyeing at room temperature for 22 h.

### 2.5. Microscope Equipment and Imaging

After staining, the embryos and larvae were washed in PBS and stored in 70% ethanol/H_2_O. Prior to imaging, the samples were transferred to a slide, 70% ethanol/H_2_O was removed, and the embryos and larvae were incubated in 90% glycerol/H_2_O for a few minutes for equilibration. After positioning the sample for the desired focal plane, images of the visualized gene expression pattern of embryos and larvae were taken under a stereomicroscope (Leica MZFLIII, Leica, Wetzlar, Germany) with the Nikon DS-Fi3 microscope camera system (Nikon, Düsseldorf, Germany) and the NIS-Elements D software version 5.11.01 64-bit.

### 2.6. Cryosections of Stained Embryos and Larvae

For detailed evaluation of the expression patterns of Ataxin-2 and Ataxin-2-like genes, the stained embryos and larvae were incubated overnight in a freshly prepared 30% sucrose/PBS solution (3 g sucrose in 10 mL PBS) after whole-mount in situ hybridization. The tissues were then incubated for one hour in a 30% sucrose/OCT solution (OCT = Polyfreeze Tissue Freezing Medium, Polysciences Europe GmbH, Hirschberg an der Bergstraße, Germany), divided into embedding molds and stored in OCT for 15 min. After a 15 min equilibration period, the embryos were aligned. Finally, the aligned embryos were frozen at −80 °C. After several days, the frozen tissues were sectioned on a cryostat (Leica Cryostat CM3050 S). The sections (10 μm) were mounted on slides, covered with PBS and coverslips, and photographed under a microscope (LEICA MZFLIII) (camera: Nikon DS-Fi3 (Nikon, Düsseldorf, Germany), program: NIS-Elements D software version 5.11.01 64-bit.

## 3. Results

### 3.1. The Proteins of the ATXN2 and ATXN2L Families Are Phylogenetically Conserved in Vertebrate Model Organisms

**Figure 1:**
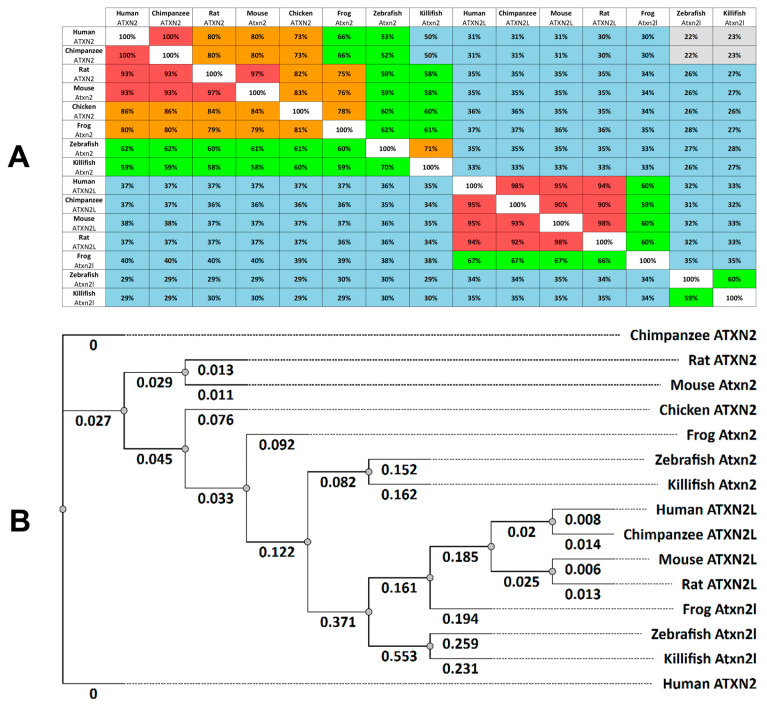
ATXN2 and ATXN2L proteins are highly conserved in vertebrates. (**A**) Multiple Sequence Alignment Distance Matrix, indicated for ataxin-2 proteins (*Homo sapiens:* Q99700.2; *Pan-troglodytes:* PNI64641.1; *Rattus norvegicus:* NP_001406666.1; *Mus musculus:* NP_033151.3; *Gallus gallus:* XP_015131043.2; *Xenopus tropicalis:* XP_002937742.3; *Danio rerio:* NP_001121821.1; *Nothobranchius furzeri:* XP_015828412.2) and ataxin-2-like proteins (*Homo sapiens:* NP_009176.2; *Pan troglodytes* XP_016784194.1; *Rattus norvegicus:* NP_001123569.2; *Mus musculus:* NP_001348416.1; *Xenopus tropicalis:* XP_031748418.1; *Danio rerio:* NP_997849.3; *Nothobranchius furzeri:* XP_070401698.1). The similarities between proteins from different species are expressed as a percentage. The colour coding indicates the similarities: red (91–100%), orange (71–90%), green (51–70%), blue (26–50%) and grey (0–25%). (**B**) The phylogenetic tree (cladogram) of ataxin-2 and ataxin-2-like amino acid sequences was derived using the PHYLIP Neighbour Joining method (Unipro UGENE software analysis tool; Tree Formation Method: PHYLIP’s Neighbor-Joining; Distance matrix model: Jones-Taylor-Thornton).

Functional studies of cell functions often require animal models to elucidate normal or pathological cell processes. Comparative analyses of gene expression domains and protein properties help to validate the role of ataxin-2 proteins in different species. Based on entries in the online databases of Ensembl and the National Center for Biotechnology Information (NCBI) (Table S1), we have identified similarities between the human proteins ATXN2 (Q99700.2) and ATXN2L (NP_009176.2) with homologues in zebrafish and other vertebrates (chimpanzees, rats, mice, frogs, and killifish, Figure 1A). Using the bioinformatic analysis software toolkit Unipro UGENE version 52.1 (https://ugene.net/) [48], we compared the human ATXN2 protein (Q99700.2) with 1313 amino acids (aa) and found high similarities with the ATXN2 orthologues in chimpanzees (100%), rats (93%), mice (93%), chickens (86%), frogs (80%), and to a lesser extent in zebrafish (62%) and killifish (59%).

A comparison of human ATXN2 (Q99700.2) with its human paralogue ATXN2L (NP_009176.2) shows that the ATXN2L protein is smaller (1075 aa). There is a 30% similarity between the two proteins. A comparative analysis of the amino acid sequence of the human ATXN2L protein with the other vertebrate paralogues shows a high identity rate between mammalian proteins (chimpanzee 95%, mouse 95%, rat 94%) and a lower identity to frogs (67%). Since birds reportedly have only one copy of ATXN2, there is no ATXN2L for chickens in the database. Compared to human ATXN2, the Atxn2l protein of the killifish shows a similarity of 34%, which is very similar to the orthologous Atxn2l of the zebrafish (33%) (Figure 1A).

Next, we analyzed the phylogenetic tree relationship of human ATXN2 and ATXNL proteins to those of other vertebrates (Figure 1B). The phylogenetic analysis was again performed using the bioinformatics analysis tool Unipro UGENE. The same amino acid sequences of the specified species (Table S1) were used as entries. Our phylogenetic analysis (tree construction method: PHYLIP Neighbor Joining; distance matrix model: Jones-Taylor-Thornton) shows a cluster for ATXN2 proteins and a more distantly related cluster for ATXN2L proteins (Figure 1B). The cladogram confirms the identity of ATXN2 in hominids (humans, chimpanzees), followed by rodents (rats, mice) with a greater distance to frogs and finally fish (zebrafish and killifish). Similar distance relationships are also found for ATXN2L clusters in these vertebrates. In general, phylogenetic analysis of ATXN2 and ATXN2L proteins in the selected vertebrates shows that the phylogenetic relationship between hominids (humans, chimpanzees), rodents (rats and mice), and fish (zebrafish and killifish) indicates a tree relationship between the selected classes. The fish proteins Atxn2 and Atxn2l are the proteins most distantly related to their human orthologues in evolutionary terms (Figure 1B).

### 3.2. The Zebrafish Locus Atxn2 Does Not Show Synteny in the Arrangement of Genes Compared to the Human ATXN2 Locus

**Figure 2:**
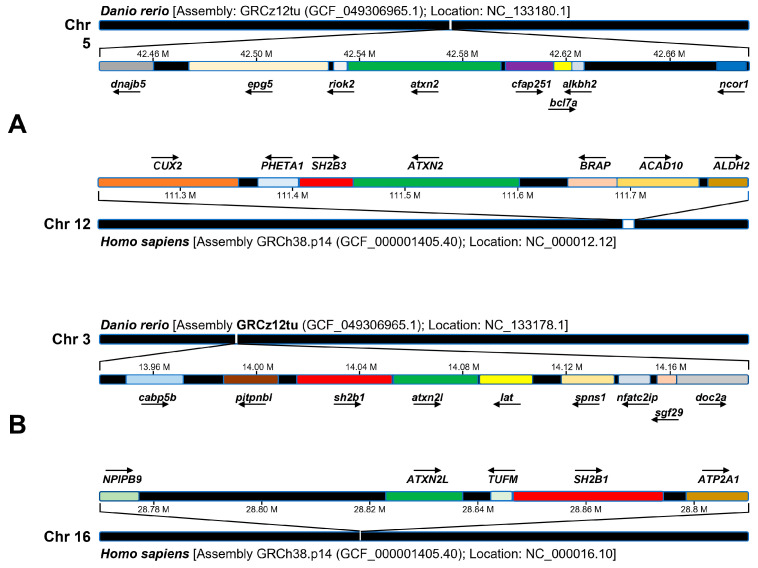
Synteny analysis of cytogenetic sites of zebrafish *atxn2*, human *ATXN2*, zebrafish *atxn2l* and human *ATXN2L*. Schematic representation of the results of the NCBI and Ensembl database analysis. (**A**) Zebrafish *atxn2* is located on chromosome 5 (reverse strand), human *ATXN2* gene on human chromosome 12q24.12 (reverse strand). (**B**) Zebrafish *ATXN2L* is located on chromosome 3 (forward strand), human *ATXN2L* on human chromosome 16p11.2 (forward strand). A comparative synteny analysis of the *ATXN2* locus of the zebrafish shows no similarity to the human *ATXN2* genome region in humans. In comparison, there is a single gene in the flanking region of the zebrafish *atxn2l,* which contains the genomic locus of *sh2b1*, that exhibits conserved synteny with a genomic region containing the *SH2B1* and *ATXN2L* genes in humans. Arrows pointing to the right indicate genes on the forward strand, arrows pointing to the left indicate genes on the reverse strand.

The study of synteny can provide insight into how the genome changes over the course of evolution. We performed a comparative synteny analysis of the zebrafish gene *atxn2* and the *atxn2-like* gene with their human orthologues (Figure 2). Using the NCBI gene database, we compared the arrangement of the genes flanking the zebrafish gene *atxn2* on chromosome 5 (*Danio rerio*: Assembly: GRCz12tu (GCF_049306965.1); location: NC_133180.1) with the arrangement of the genes of the human *ATXN2* locus on chromosome 12 (*Homo sapiens*: Assembly GRCh38.p14 (GCF_000001405.40); position: NC_000012.12). The arrangement of the flanking genes of the *atxn2* locus in zebrafish differs completely from the neighboring genes of the human orthologous region. None of the flanking gene loci are identical between the two species (Figure 2A).

Next, we compared the assemblies of the genes of the zebrafish locus *atxn2l* on chromosome 3 (*Danio rerio*: assembly GRCz12tu (GCF_049306965.1); position: NC_133178.1) with those of the *ATXN2L* locus on chromosome 16 (*Homo sapiens*: assembly GRCh38.p14 (GCF_000001405.40); position: NC_000016.10). Only one locus, the *sh2b1* gene in zebrafish, is identified as the *SH2B1* gene in the arrangement of the human *ATXN2L* locus (Figure 2B). The synteny of the *sh2b1* and *atxn2l* loci indicates an evolutionary relationship between the two gene arrangements in humans and zebrafish, in contrast to the *atxn2* gene in zebrafish, for which no syntenic regions were found in the chromosomal region of human *ATXN2*.

### 3.3. Structure of the Zebrafish Genes Atxn2 and Atxn2l and the Proteins They Encode

Prior to the expression analysis of the *atxn2* and *atxn2l* genes in zebrafish, we performed a bioinformatic analysis of both genes at the genome and transcript level and compared the proteins and their domains.

**Figure 3:**
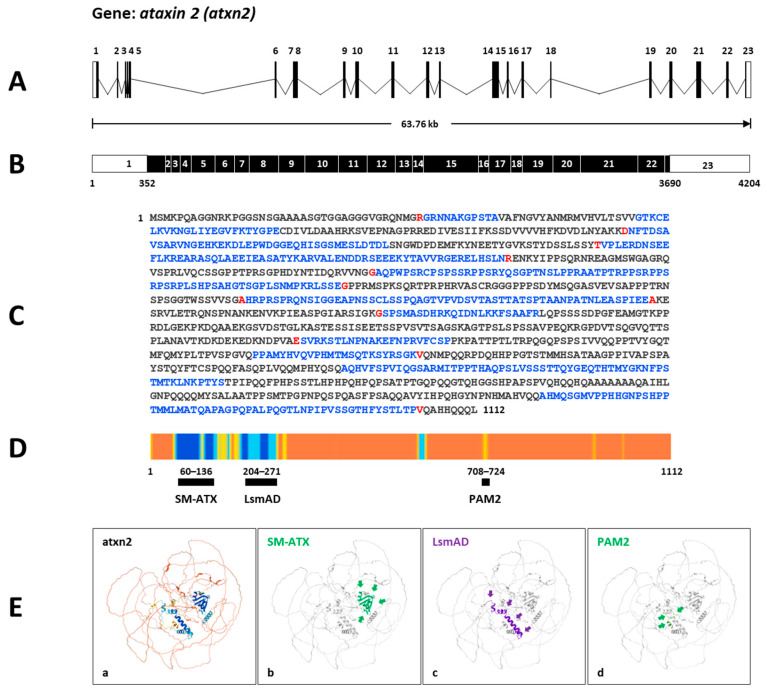
*Atxn2* gene and protein in zebrafish. (**A**) Schematic presentation of the structure of the *atxn2* gene on the inverted strand of chromosome 5. The gene covers a genomic region of 63.76 kb and contains 23 coding exons. (**B**) The derived mRNA (Ensembl: transcript ID ENSDART00000083656.5 *atxn2-201*) has 4204 nucleotides with an open reading frame (black box) between the nucleotide 352 in exon 1 and the nucleotide 3690 in exon 23. (**C**) The translated Atxn2 protein contains 1112 amino acids (translation ID: ENSDARP00000078091.4). The amino acids from each exon are identified by different colors (black: an exon; blue: another exon; red: another exon residue overlaps the splice site). (**D**) The Ensembl *atxn2* transcript matches the UniProt identifier A2CF3, which indicates the AlphaFold confidence for identified domains in zebrafish Atxn2. Alphafold generates a Pro-Residue Confidence Score (PLDDT) between 0 and 100: dark blue: very high (pLDDT > 90); light blue: confident (90 > pLDDT > 70; yellow: low (70 > pLDDT > 50); orange: very low (pLDDT < 50). Some regions with low pLDDT may be unstructured in isolation. The highest values were identified in the N-terminal region of the zebrafish Atxn2. (**E**) (**a**)The AlphaFold predicted model of the Atxn2 protein of the Ensembl zebrafish ENSDARP00000078091 indicates (**b**) an SM-ATX domain, (**c**) an LsmAD domain, and (**d**) a PAM2 motif. The protein domains are highlighted and marked by arrows.

Firstly, according to the ZFIN database, the zebrafish gene *atxn2* (https://zfin.org/ZDB-GENE-060526-217; URL accessed on 25 August 2025) spans a 63.76 kb section of the genome on the reverse strand of chromosome 5: 41,645,479–41,709,234 (Figure 3A). Transcript ID ENSDART00000083656.5 (*atxn2-201*) is a product of the gene ENSDARG00000052897. The mRNA contains 4204 nucleotides and is transcribed from 23 coding exons, with the open reading frame beginning in exon 1 and ending in exon 23 (Figure 3B). The translated protein ID (ENSDARP00000078091.4) contains 1112 aa (Figure 3C). Predicted protein domains and motifs from InterPro (https://www.ebi.ac.uk/interpro/; URL accessed on 25 August 2025) and the Pfam database [50,51] assign three domains to zebrafish Atxn2 (SM-ATX domain: position 62–136; LsmAD domain: position 204–271; PAM2 motif: position 708–271) (Figure 3D). The UniProt identifier A2CF3, which matches the Ensembl transcript of *atxn2*, shows the predicted domains with high confidence in the AlphaFold model (Figure 3E).

**Figure 4:**
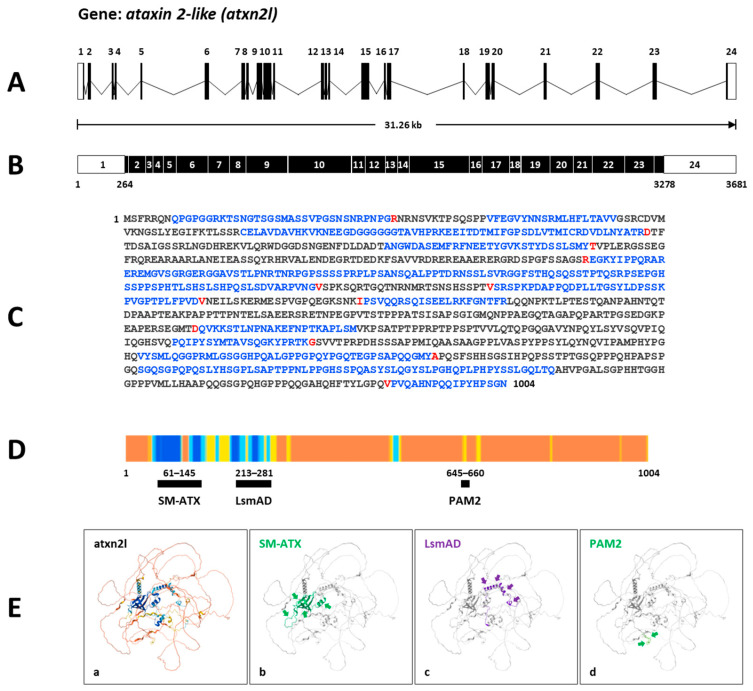
*Atxn2l* gene and protein in zebrafish. (**A**) Schematic presentation of the structure of the *atxn2l* gene on the forward strand of chromosome 3. The gene covers a genomic region of 31.26 kb and contains 24 coding exons. (**B**) The derived mRNA (Ensembl: transcript ID ENSDART00000133168.3 *atxn2-201*) has 3681 nucleotides with an open reading frame (black boxes) between the nucleotide 264 in exon 1 and the nucleotide 3278 in exon 24. (**C**) The translated Atxn2l protein contains 1004 amino acids (translation ID: ENSDARP00000115477.1). The amino acids from each exon are identified by different colors (black: an exon; blue: another exon; red: another exon residue overlaps the splice site). (**D**) The Ensembl *atxn2l* transcript matches the UniProt identifier F1QA42, which displays the AlphaFold confidence for identified domains in the zebrafish Atxn2l. Alphafold generates a Pro-Residue Confidence Score (PLDDT) between 0 and 100: dark blue: very high (pLDDT > 90); light blue: confident (90 > pLDDT > 70; yellow: low (70 > pLDDT > 50); orange: very low (pLDDT < 50). Some regions with low pLDDT may be unstructured in isolation. The highest values were identified in the N-terminal region of the zebrafish Atxn2l. (**E**) (**a**) The AlphaFold-predicted model of the Atxn2l protein of the Ensembl zebrafish ENSDARP00000115477.1 indicates (**b**) an SM-ATX domain, (**c**) an LsmAD domain, and (**d**) a PAM2 motif. The protein domains are highlighted and marked by arrows.

Secondly, the zebrafish gene *atxn2l* (https://zfin.org/ZDB-GENE-030131-3246; URL accessed on 25 August 2025) spans a genomic segment of 31.26 kb on the forward strand of chromosome 3: 15,394,428–15,425,689 (Figure 4A). Transcript ID ENSDART00000133168.3 (*atxn2l-201*) is a product of the gene ENSDARG00000011597. It contains 3681 nucleotides and is transcribed from 24 exons, with the open reading frame beginning in exon 1 and ending in exon 24 (Figure 4B). The translated protein ID (ENSDARP00000115477.1) contains 1005 aa (Figure 4C). InterPro assigns three domains to zebrafish Atxn2l (SM-ATX domain: position 61–145; LsmAD domain: position 213–281; PAM2 motif: position 645–660) (Figure 4D). The UniProt identifier F1QA42 indicates an AlphaFold model with a high confidence value showing the predicted domains in zebrafish Atxn2l (Figure 4E).

**Figure 5:**
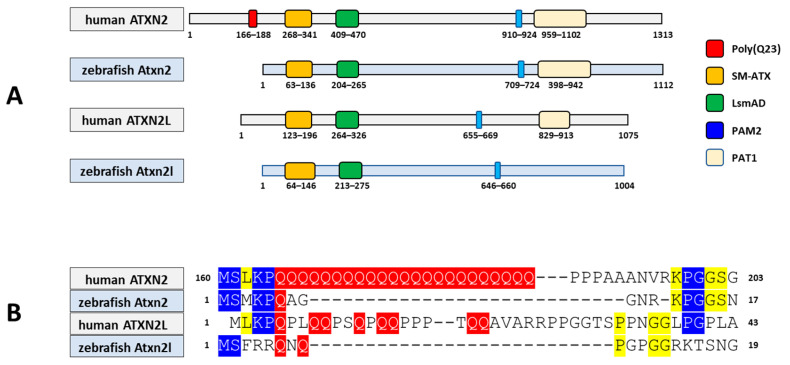
The Atxn2 and Atxn2l proteins of the zebrafish share conserved domains with their human homologues. (**A**) Schematic representation of proteins representing conserved protein domains (CDD) using the NCBI Conserved Domain Database (CDD) search. For the human protein ATXN2–218 (ENSP00000446576.2, 1313 aa, UniProt Match Q99700-1), only concise results of the CD search are presented; Zebrafish protein Atxn2-201 (ENSDARP00000078091.4, 1112 aa, UniProt Match A2CF31); human protein ATXN2-like-202 (ENSP00000338718.4, 1075 aa, UniProt Match Q8WWM7-1) and zebrafish protein Atxn2-like-201 (ENSDARP00000115477.1, 1004 aa, UniProtMatch F1QA42). The CD search for human ATXN2 reveals four conserved domains (SM-ATX, LsmAD, PAM2 and topoisomerase II-associated protein PAT1). The poly(Q23) domain in human ATXN2 is not displayed by the CD search, but the position (red box) is displayed. Human ATXN2L, zebrafish Atxn2 and Atxn2l do not have a polyQ domain. The PAT1 domain is not found in the zebrafish Atxn2l. (**B**) Although the polyQ stretch is detectable only in human ATXN2, the flanking amino acid residues are conserved at the same site as the corresponding glutamine in the human paralogue ATXN2L and the zebrafish orthologues Atxn2 and Atxn2l. Colour markings indicate the number of amino acid matches in the four proteins. The polyglutamine domain and the respective positions of the glutamines are marked in red. Three matches are marked in blue, two matches in yellow.

Thirdly, we compared the predicted protein domains of the zebrafish protein Atxn2 (NP_001121821.1; 1112 aa) and the protein Atxn2l (NP_997849.3; 1004 aa) with their human orthologues ATXN2 (Q99700.2; 1313 aa) and ATXN2L (NP_009176.2; 1075 aa). Using the NCBI database, we employed the CD algorithm [49] to display conserved domains in the proteins. The output files (database: CDSEARCH/oasis_pfam) were adjusted for concise results with the highest E-values (E-value threshold: 0.01), a composition-based adjustment (yes), and a low complexity filter (yes) (Figure 5A). The results show three domains, as expected for the human ATXN2 protein: SM-ATX (Ataxin 2 SM domain: aa 268–341, accession number pfam 14438); LsmAD (LsmAD domain: aa 409–470, accession number pfam06741); PAM2 (Ataxin-2 C-terminal region: aa 910–924, accession number pfam07145) and, in addition, a fourth domain PAT1 (Topoisomerase II-associated protein PAT: aa 959–1102, accession number pfam09770).

Identical domains were identified in the Atxn2 protein of zebrafish: SM-Atx (aa 63–136), LsmAD (aa 204–265), PAM2 (aa 709–724), and PAT1 (aa 766–911). These four domains were also identified in human ATXN2L: Sm-ATX (aa 123–196), LsmAD (aa 264–326), PAM2 (aa 655–669), and PAT1 (aa 829–913). In contrast, only three domains were identified in the zebrafish Atxn2l protein: SM-Atx (aa 64–146); LsmAD (aa 213–275); PAM2 (aa 646–660). The PAT1 domain is not indicated for zebrafish Atxn2l. This also applies to the polyQ domain with 23 glutamine residues, which is not found in human ATXNL or in zebrafish Atxn2 and Atxn2l. It should be noted that the polyQ domain of human ATXN2 was not listed in the CD search results and is indicated separately in Figure 5A (red box). Interestingly, despite the lack of a glutamine stretch in these three proteins, conserved amino acid sequences flank the glutamine residue sites in these proteins (Figure 5B).

### 3.4. Expression Analysis of Zebrafish Atxn2 and Atxn2l Using RT-PCR

**Figure 6:**
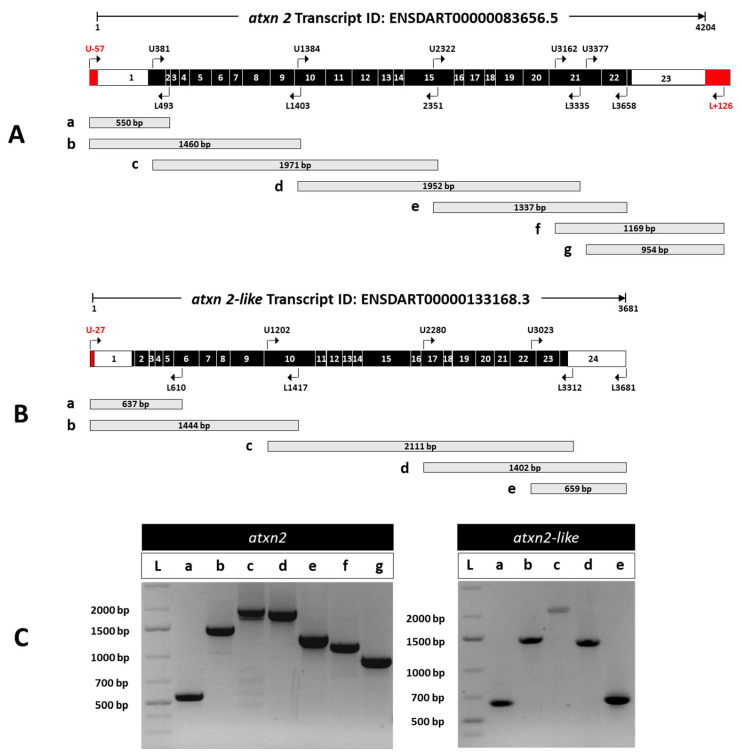
Experimental detection of *atxn2* and *atxn2l* transcripts by RT-PCR in zebrafish. (**A**) Schematic representation of *atxn2* (transcript ID: ENSDART00000083656.5). Along the 4204 nucleotides (nt) sequence, exon-spanning primer pairs are displayed with specified positions in the transcript and the expected length of the PCR products. The coding exons (black boxes) are flanked by 5′-UTR and 3′-UTR (white boxes), including extended genomic sequences at both ends (red boxes). The upper primer U-57 binds at the 5′–end 57 nt before the predicted nucleotide 1 in exon 1 and L + 126 binds 126 nucleotides after the predicted 3′–end of the 4204 nt long transcript. (**B**) *atxn2l* (transcript ID: ENSDART00000133168.3) with the indicated positions of the primer pairs used in the transcript and the expected length of the PCR products are displayed along the 3681 nt sequence. The coding exons (black boxes) are flanked by 5′-UTR and 3′-UTR (white boxes), including an extended exon 1 sequence at the 5′-end (red box). The upper primer U-27 binds to a nucleotide of the 5′-exon sequence 27 before the predicted first nucleotide of the transcript. (**C**) RT-PCR products of *atxn2* (left) and *atxn2l* (right) amplicons are shown. The fragment sizes are as expected. The primer used in the extended first and last exon of *atxn2* and in the extended first exon of *atxn2l* confirms the extension of the *atxn2* and *atxn2l* transcripts as indicated. The respective positions and expected lengths of the amplification products are shown schematically for *atxn2* in (**A**) (a, b, c, d, e, f, g) and for *atxn2l* in (**B**) (a, b, c, d, e). (**C**) shows the corresponding RT-PCR products of *atxn2* (left panel) and *atxn2l* (right panel) after gel electrophoresis, labelled with the same markers.

Based on the ZFIN database, the Ensembl transcript ID according to ENSDART00000083656.5 (*atxn2-201*) contains 4204 nucleotides. Using the Ensembl transcript chromosome ENSDART00000083656.5: GRCz11:5:1:72500376: 1, we performed a BLAST (version BLAST+ 2.17.0: 22 July 2025) search in NCBI in the refseq_rna database for *Danio rerio* (taxid: 7955) and found an annotated *atxn2* mRNA sequence match with a shorter transcript (NM_001128349) comprising 3339 identical nucleotides. An additional 28 transcript variants (X1–X28) were identified by bioinformatic search analysis with an identity of more than 97.8 percent to transcript ENSDART00000083656.5 (*atxn2-201*). All predicted *Danio rerio atxn2* variants had extended sequences at both the 5′– and 3′–ends and one or two gaps between them. We used the sequence data from genomic DNA (CR848794.6) at the 5′ end of the first exon and the sequence of the predicted *atxn2* transcript variant X1 (XM_009301663.5) at the 3′ end to extend the ENSDART00000083656 transcript at both ends and select suitable primer pairs for RT-PCR, as shown in the red boxes in Figure 6A.

We used a similar approach for *atxn2l*. According to the ZFIN database, the Ensembl transcript ID ENSDART00000133168.3 (*atxn2l-201*) has a length of 3681 nucleotides. A BLAST search in NCBI in the refseq_rna database for *Danio rerio* (taxid: 7955) yielded an annotated *atxn2l* mRNA transcript (NM_212684.3) with a length of 3607 nucleotides, which overlaps with four predicted *Danio rerio atxn2l* transcript variants (X1–X4). We used the sequence data from genomic DNA (BX784026.17) to extend the 5′ end of the Ensembl transcript ID ENSDART00000133168.3 (*atxn2l-201*) for primer selection for RT-PCR (red box in Figure 6B).

To verify the expression of the predicted and annotated transcripts, we performed RT-PCR using total RNA isolated from adult zebrafish bodies. After reverse transcription of the mRNA, the cDNAs of *atxn2* and *atxn2l* were amplified with primer pairs (Table S2) covering sections of the entire transcript length, as shown schematically (Figure 6A,B). The PCR amplicons for *atxn2* (Figure 6C, left panel) and *atxn2l* (Figure 6C, right panel) yielded the expected fragment sizes for the selected regions for both the *atxn2* and *atxn2l* cDNAs. Furthermore, both ends of transcript ENSDART00000083656.5 (*atxn2-201*) could be amplified at the predicted 5′ and 3′ ends, indicating an extension of the first and last exons of this transcript, at least for the selected sequence. This also applies to exon 1 in the case of the Ensembl *atxn2l* transcript ENSDART00000133168.3 (*atxn2l-201*). Overall, the experimental data confirmed the expression of the expected and predicted *atxn2* and *atxn2l* transcripts in the adult zebrafish body.

**Figure 7:**
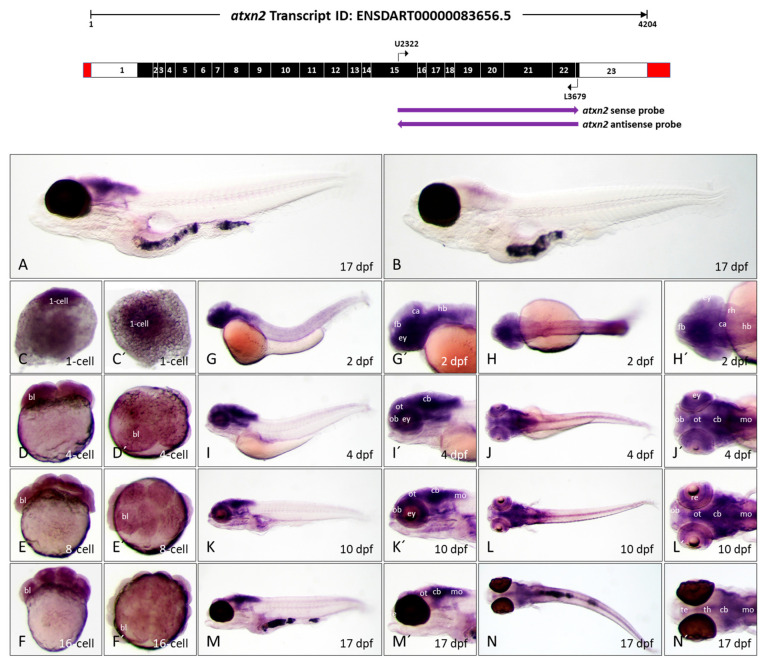
*Atxn2* expression patterns in zebrafish embryos and larvae. In situ hybridization was performed on embryos and larvae of the *brass* line (1-cell—10 dpf) and the *casper* line (17 dpf). (**A**) Expression domains of *atxn2* in the whole larva (17 dpf) detected by antisense probes in the coding region of the transcript, denoted by the left arrow below the schematized transcript (upper image). (**B**) Sense probes (indicated by right arrow, upper image) show a weak background coloration throughout the larva (17 dpf). The images (**C**–**F**) show side views and (**C′**–**F′**) show views from the animal pole of *atxn2* expression from the zygote to the embryos in the 16-cell stage. The images (**G**,**I**,**K**,**M**) show side views and (**H**,**J**,**L**,**N**) dorsal views of whole embryos (2 dpf, 4 dpf) and larvae (10 dpf and 17 dpf). Enlarged head sections of lateral pictures are shown in (**G′**,**I′**,**K′**,**M′**) and of dorsal pictures in (**H′**,**J′**,**L′**,**N′**). Abbreviations: bl (blastomere), ca (cerebellar anlage), cb (cerebellum), ey (eye), fb (forebrain), hb (hindbrain), mo (medulla oblongata), ob (olfactory bulb), ot (optic tectum), re (retina), rh (rhombencephalon), te (telencephalon), th (thalamus).

**Figure 8:**
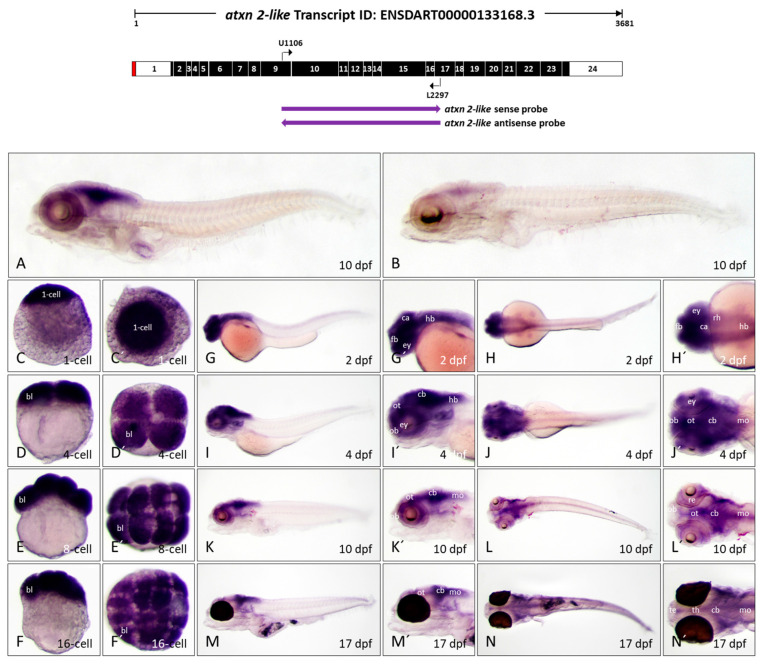
*Atxn2l* expression pattern in zebrafish embryos and larvae. In situ hybridization was performed on embryos and larvae of the *brass* line (1 cell-10 dpf) and the *casper* line (17 dpf). (**A**) Expression domains of *atxn2l* in the whole larva (10 dpf) detected by antisense probes in the coding region of the transcript, indicated by the left arrow below the schematized transcript (upper image). (**B**) Sense probes (indicated by right arrow, upper image) indicate weak background coloration throughout the larva (10 dpf). The images (**C**–**F**) show lateral views and (**C′**–**F′**) show views from the animal pole of *atxn2l* expression from the zygote to embryos in the 16-cell stage. The images (**G**,**I**,**K**,**M**) show lateral views and (**H**,**J**,**L**,**N**) show dorsal views of whole embryos (2 dpf, 4 dpf) and larvae (10 dpf and 17 dpf). Enlarged head sections from the lateral view are shown in (**G′**,**I′**,**K′**,**M′**) and from the dorsal view in (**H′**,**J′**,**L′**,**N′**). Abbreviations: bl (blastomere), ca (cerebellar anlage), cb (cerebellum), ey (eye), fb (forebrain), hb (hindbrain), mo (medulla oblongata), ob (olfactory bulb), ot (optic tectum), re (retina), rh (rhombencephalon), te (telencephalon), th (thalamus).

Interestingly, there are almost no expression patterns of *atxn2* and *atxn2l* in zebrafish embryos and larvae in the literature. According to database entries in ZFIN (ZFIN.org), no whole-mount in situ hybridization (WISH) images are documented for zebrafish embryos, with the exception of the trunk region of 2 dpf (days post-fertilization) to 4 dpf old embryos [53]. To fill this knowledge gap, we investigated the expression of *atxn2* and *atxn2l* in zebrafish during early embryonic development and in the larval stage. We tested the specificity of different antisense probes in comparison to the corresponding sense probes and selected the antisense probe that produced the weakest sense signal for each *atxn2* and *atxn2l* transcript for further experiments. WISH was performed at different stages of embryonic development (1-cell, 4-cell, 8-cell, and 16-cell stages, 2 dpf, and 4 dpf) and at two larval stages (10 dpf and 17 dpf). The *brass* zebrafish line was used for all developmental stages from 1 cell to 10 dpf, and the *casper* line was used for 17 dpf-old larvae (Figure 7 and Figure 8).

#### 3.4.1. Atxn2 Transcripts Are Expressed from Early Embryonic to Late Larval Stages in Zebrafish

To analyze the expression pattern of the *atxn2* gene during zebrafish development, a 3′-terminal region of the *atxn2* transcript ID: ENSDART00000083656.5 was selected for the generation of sense and antisense probes for WISH. The probe (1358 nucleotides) spans the transcript region encoded between exon 15 and exon 23 (schematically shown in Figure 7, upper panel). The specificity of the antisense probes for *atxn2* was first successfully tested in 17 dpf whole-mount larvae (Figure 7A). The sense probe (control) showed slight background staining (Figure 7B).

*Atxn2* transcripts are already present in the zygote (1-cell stage) and are also detected after the first cell divisions in the 4-, 8-, and 16-cell stages (Figure 7, sagittal views C–F, views from the animal pole C’–F’). In the 2-day-old zebrafish embryo, strong expression of *atxn2* can be observed in the brain region and in the anterior part of the body (Figure 7G,G’,H,H’), with ubiquitous but weaker expression in the trunk and tail region. In the 4 dpf zebrafish embryo, the main expression of *atxn2* is limited to the brain region, including the telencephalon, tectum opticum, rhombencephalon, cerebellum, medulla oblongata, and the retina of the eyes (Figure 7I,I’,J,J’). In the 10-day-old zebrafish larva, the expression pattern of *atxn2* is more strongly restricted to the posterior brain region. The expression strength of *atxn2* remains unchanged in the optic tectum, cerebellum, medulla oblongata, and spinal cord, but becomes significantly weaker in the anterior telencephalon and posterior part of the body (Figure 7K,K’,L,L’). The expression domains of *atxn2* in the 17-day-old zebrafish larva are almost identical to those of the 10 dpf larva, but the domains in the posterior brain regions, especially in the optic tectum, cerebellum, medulla oblongata, and spinal cord, are more pronounced than in the 10-day-old larva (Figure 7M,M’,N,N’). Since the *casper* line was used for the 17 dpf zebrafish larvae, *atxn2* expression in the retina cannot be shown due to the darkly pigmented eyes of this line.

In summary, the WISH experiments showed that *atxn2* transcripts are already detectable in the zygote and in embryonic cells after the first cell divisions, suggesting that *atxn2* is a maternally expressed gene. *Atxn2* is expressed in several brain regions during embryonic and larval development in zebrafish. With increasing developmental age, the *atxn2* expression domains are increasingly limited to the midbrain and hindbrain regions, the medulla oblongata and the spinal cord.

#### 3.4.2. Atxn2l Shares Expression Domains with Atxn2 from Early Embryonic to Late Larval Stage in Zebrafish

To detect *atxn2l* expression during zebrafish development, a segment of transcript ENSDART00000133168.3 was chosen to create antisense and sense probes for WISH, as illustrated in Figure 8 (top). The probe region (1192 nucleotides) detects transcripts between exon 9 and exon 17 (schematically shown in Figure 8, upper image). An antisense probe comprising 1192 nucleotides, specific to *atxn2l*, was utilized on whole-mount larvae at 10 days post-fertilization, as depicted in Figure 8A. The same transcript region for the sense probe (control) showed only minimal background staining (Figure 8B). The WISH results showed that *atxn2l* transcripts are also detected in early embryonic developmental stages. The zygote (1-cell) and all embryonic cells after the first cell divisions have high *atxn2l* transcript levels, as evidenced by strong staining of blastomeres at the 2-cell, 4-cell, and 16-cell stages (Figure 8, sagittal views C–F, views from the animal pole C’–F’). In the 2-day-old zebrafish embryo, a high expression of *atxn2l* can be observed in the brain and in the medulla oblongata. Low expression of *atxn2l* is visible in the spinal cord, while almost no expression is detectable in the remaining trunk and tail tissues (Figure 8G,G’,H,H’). In 4-dpf zebrafish embryos, *atxn2l* is primarily expressed in the telencephalon, optic tectum, rhombencephalon, cerebellum, and medulla oblongata. In addition, high concentrations of *atxn2l* transcripts are also found in the retina of the eyes (Figure 8I,I’,J,J’). In the 10-day-old zebrafish embryo, the expression pattern is even more pronounced. The expression domain within the telencephalon is diminished. High expression is confined to the regions of the optic tectum, the cerebellum, the medulla oblongata, and the eyes (Figure 8K,K’,L,L’). The same expression domains of *atxn2l* exist in the 17-day-old zebrafish larva. However, *atxn2l* is predominantly expressed in the posterior regions of the brain, with significantly high expression levels in the optic tectum, cerebellum, and medulla oblongata. The spinal cord in the posterior part of the body also weakly expresses *atxn2l*, which is more visible in the dorsal view (Figure 8M,M’,N,N’). Since the eyes of the 17 dpf old zebrafish embryos of the *casper* line are highly pigmented, no conclusions could be drawn about the expression of *atxn2l* in the retina of the eyes at this stage of development.

Overall, the WISH experiments on *atxn2l* transcripts show a high level of maternal transcripts that are already present in the zygote and remain in the blastomeres even after the first divisions. *Atxn2l* expression domains are initially found in all regions of the brain, the medulla oblongata and the spinal cord during embryonic and larval development in zebrafish. With increasing developmental age, the expression of *atxn2l* decreases in the telencephalon, but remains high in certain regions of the midbrain and hindbrain, the medulla oblongata and the spinal cord.

#### 3.4.3. Cryosections Confirm the Expression Domains of Atxn2 and Atxn2l in Different Brain Regions of Zebrafish Embryos and Larvae

**Figure 9:**
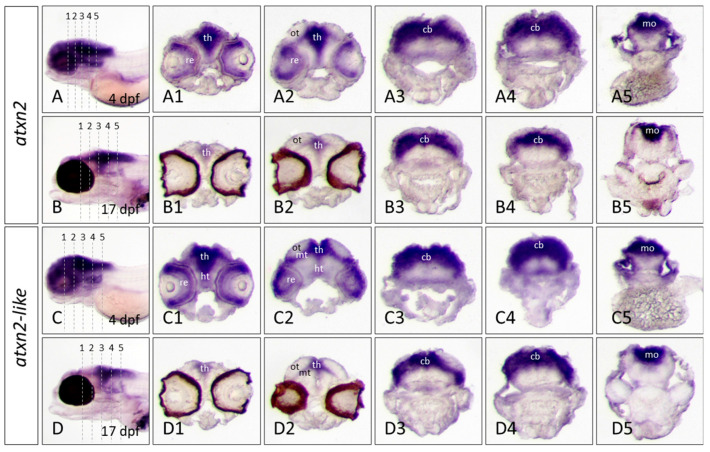
Expression domains of *atxn2* and *atxn2l* in cryosections of zebrafish embryos and larvae. In situ hybridization was performed with embryos of the *brass* line (4dpf) and larvae of the *casper* line (17 dpf). Expression domains of *atxn2* were detected with antisense probes for the coding region of the transcript (shown in the upper fields of Figure 7 and Figure 8). (**A**–**D**) show the position of transverse cryosections (10 μm) of five different regions of the head (indicated in the left panels). Serial rostral to caudal views of the head region are shown for *atxn2* expression domains at developmental stage of 4 dpf (**A1**–**A5**) and for the *casper* larva at 17 dpf (**B1**–**B5**). *Atxn2l* expression domains can be seen in serial rostral to caudal views of the head region in the brass embryo at developmental stage of 4 dpf (**C1**–**C5**) and the *casper* larva at 17 dpf (**D1**–**D5**). Abbreviations: cb (cerebellum), ht (hypothalamus), mo (medulla oblongata), mt (midbrain tegmentum), ot (optic tectum), re (retina), th (thalamus).

In situ hybridization of a whole embryo can also lead to overstaining of highly expressed tissue. To visualize the expression domains of the *atxn2* and *atxn2l* genes more precisely, cryosections of the head region of already stained embryos (4 dpf) and larvae (17 dpf) were prepared (Figure 9). The position of the cryosections (10 μm) is indicated for each embryo (2 dpf and 4 dpf) and larva (10 dpf and 17 dpf) by numbers (1–5) in an anterior to posterior order (Figure 9A–D). The expression domains of *atxn2* are clearly visible in different regions of the brain of the 4 dpf old embryo of the *brass* line. *Atxn2* is expressed in the retina, thalamus, hypothalamus, optic tectum, and cerebellum. The level of expression in the hypothalamus is lower than in the thalamus, optic tectum, and cerebellum (Figure 9(A1–A5)). The tissue sections of a 17 dpf old zebrafish embryo of the *casper* line show that *atxn2* is expressed in the same brain regions as in a 4 dpf old embryo. However, the expression strength of *atxn2* is reduced and only more pronounced in the thalamus and optic tectum (Figure 9(B1,B2)) but is still high in the cerebellum and medulla oblongata (Figure 9(B3–B5)). In 4dpf embryo tissue sections, *atxn2l* and *atxn2* are both expressed in the same brain regions. *Atxn2l* is expressed particularly in the thalamus, hypothalamus, optic tectum and cerebellum of a 4-day-old zebrafish embryo (Figure 9(C1–C5)). In a 17-day-old zebrafish, the *atxn2l* expression domains are similar to those of a 4 dpf old embryo. However, more restricted domains are found in the thalamus and the optic tectum (Figure 9(D1–D2)), compared to the cerebellum and the medulla oblongata, which still have high levels of expression (Figure 9(D3–D5)). Due to the natural eye pigmentation of the *casper* line, the retina cannot be determined with certainty as a possible expression site of *atxn2* and *atxn2l* at this stage of development of 17 dpf old larvae.

### 3.5. RT-PCR Analysis of Atxn2 and Atxn2l Expression Levels in Whole-Body Zebrafish

**Figure 10:**
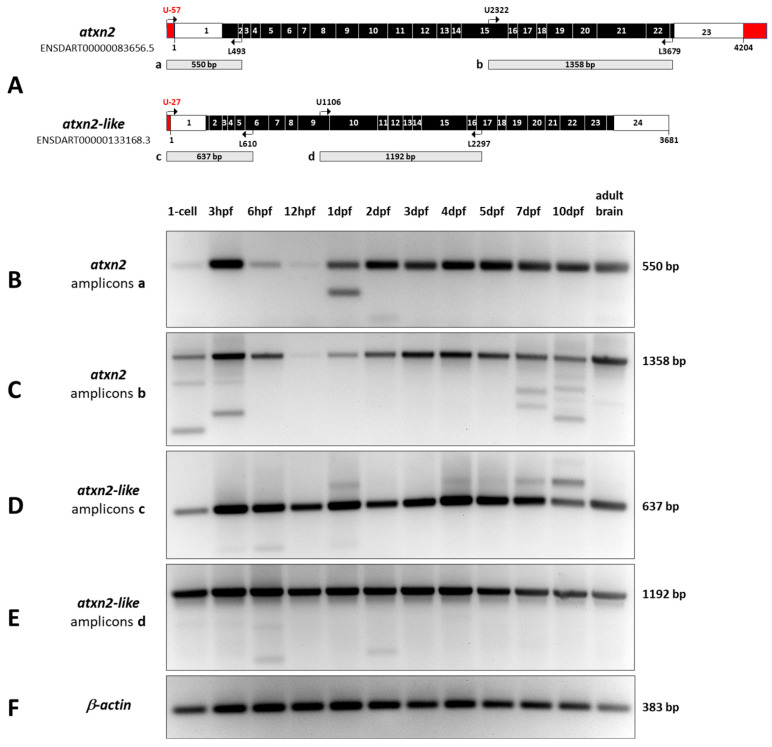
Expression levels of *atxn2* and *atxn2l* during zebrafish development. Total RNA was isolated from embryos and larvae at different stages of development (1-cell zygote, 3 hpf, 6 hpf, 12 hpf, 1 dpf, 2 dpf, 3 dpf, 4 dpf, 5 dpf, 7 dpf and 10 dpf) and from adult zebrafish brains. The mRNA was reverse transcribed into cDNA. (**A**) Two different gene-specific primer pairs for *atxn2* and *atxn2l* transcripts were used to amplify two different regions of the cDNAs (amplicons a, b, c, d). (**B**–**F**) RT-PCR products show developmentally regulated expression levels of *atxn2*, while expression levels of *atxnl* remain constant throughout zebrafish development. In addition to the expected amplicon sizes, fragments of different sizes indicate alternative splicing events during development, which are more pronounced in *atxn2* than in *atxn2l*. *b-actin* transcripts were amplified as a control.

The results of the in situ hybridizations showed that *atxn2* and *atxn2l* gene products are already present in the zygote and are expressed in high quantities in the neuronal structures of zebrafish embryos and larvae. In order to be able to compare the expression levels of both genes during development more precisely, we performed additional RT-PCR experiments. To this end, we isolated total RNA from whole-body embryos and larvae of zebrafish at different stages of development (1-cell, 3 hpf, 6 hpf, 12 hpf, 1 dpf, 2 dpf, 3 dpf, 4 dpf, 5 dpf, 7dpf, 10 dpf) and from the adult zebrafish brain. After reverse transcription of mRNA to cDNA, we used equal amounts of cDNA as a template for PCR using two specific primer pairs for each transcript (*atxn2:* sequence ID ENSDART00000083656.5 and *atxn2-like*: sequence ID ENSDART00000133168.3) (Table S2) to amplify two different PCR products from each of the two transcripts (schematically shown in Figure 10A).

The results of *atxn2* expression in zebrafish development are demonstrated by two different amplified PCR products, one covering the 5′-end (550 bp) and the other the 3′-region (1358 bp) of the transcript (Figure 10B,C). The *atxn2* amplicon a band (550 bp) is weak, but already detectable at the 1-cell stage (Figure 10B). A sharp increase in *atxn2* levels is observed during the blastula period (3 hpf). The amount of *atxn2* transcripts decreases significantly after gastrulation (6 hpf) and is almost undetectable at the segmentation stage (12 hpf). Starting at the pharyngula stage (1 dpf), the expression intensity gradually increases and remains at a constant level during development from the hatching period 2 dpf to 10 dpf and in the adult brain. An additional shorter band of amplicon a in the prim-5 stage (1 dpf) and a weak second short band in the long-pec stage (2 dpf) indicate splice variants at the 5′ end of the *atxn2* transcript. The *atxn2* amplicon b (1358 bp) shows an almost identical pattern to amplicon a at all stages of development (Figure 10C). Shorter splice variants are found at the 1-cell stage, in the blastula period (3 hpf), and during larval development at 7 and 10 dpf. A faint, shorter splice variant appears in the adult zebrafish brain.

The results of *atxn2l* expression in zebrafish development are illustrated by two amplified PCR products of the 5′-end (637 bp) and a middle region of the coding sequence (1192 bp) of the transcript (Figure 10D,E). The *atxn2l* amplicon c band (637 bp) (Figure 10D) shows *atxn2l* transcripts as early as the 1-cell stage and almost constant expression levels in all other developmental stages and in the brain of the adult zebrafish. Weak and shorter transcript variants are detected in the blastula (3 hpf) and gastrula (6 hpf) as well as in the pharyngula period (1 dpf). Weak longer splice variants are present at 1 dpf, 4 dpf, 5 dpf, 7 dpf, and 10 dpf. In the brains of adult zebrafish, no splicing event for the 5′-end of *atxn2l* was detected. The amplicons d, which indicate the PCR products of the *atxn2l* central region of the coding transcript, show a constant level of expression from the 1-cell stage through all developmental stages up to 10 dpf and in the adult brain (Figure 10E). Only weak and shorter splice variants are present in the 1-cell stage, gastrula (6 hpf) and pharyngula (2dpf) periods, and probably in the developmental stage of 5 dpf and 7 dpf. No splicing of the *atxn2l* amplicon d transcript was found in adult zebrafish brains. The amplicons of *b-actin* (383 bp) (Figure 10F) show constant amounts of cDNA from all tissues used for PCR as controls.

In summary, RT-PCR analyses for *atxn2* and *atxn2l* show that the expression of *atxn2* is developmentally regulated from the 1-cell stage to the pharyngula period at 1 dpf, with constant levels in later stages and in the adult brain. In contrast, the expression of *atxn2l* shows constant transcript levels across all developmental stages (1-cell to 10 dpf) and in the brain of the adult zebrafish. The results also show that *atxn2* and *atxnl* expression leads to alternative splicing events at the same developmental stages that could not be detected in the adult zebrafish brain using the selected primer pairs and transcript regions.

## 4. Discussion

*ATXN2* has been known as one of the few genes in which a single gene causes multiple diseases and/or alters different neurological disorders [54]. A broad knowledge of the roles of *ATXN2* is a prerequisite for developing new approaches for the treatment and prevention of disease symptoms. This also applies to the paralogous gene *ATXN2L* [55], which has a significant similarity to *ATXN2*.

The phylogenetic evolution of the *ATXN2* gene applies only to eukaryotes such as protists, fungi, animals, and plants, but has never been identified in prokaryotes [56]. Interestingly, although the zebrafish *Danio rerio* has become a prominent animal model organism, there is still a knowledge gap of *atxn2* and *atxn2l* in this species. In this study, we used annotated ATXN2 and ATXN2L protein sequences from the Ensembl and NCBI databases and applied the Unipro UGENE software tool to compare the protein sequences of human and other vertebrate models with the zebrafish orthologues. As expected, two groups of phylogenetic relationships were indicated for ATXN2 and ATXN2L. While the human ATXN2 protein is identical to that of chimpanzees (100%), less amino acid similarities of ATXN2 are found in orthologues of rat (93%), mouse (93%), chicken (86%), frog (80%), zebrafish (62%) and killifish (59%). The similarity of the two fish Atxn2 proteins to the human ATXN2 is still higher than between the two human paralogues. The human ATXN2 has a sequence similarity of only 37% with its own paralogue ATXN2L, which forms the second group of protein similarities between the species. The comparison between the human ATXN2L (100%) showed a similarity of the amino acid sequences: in chimpanzees (95%), mice (95%), rats (94%), frogs (67%), zebrafish (34%) and killifish (35%), indicating an overall reduced similarity of Atxn2l in the fish. A comparison with birds could not be carried out, as they have only one orthologue ATXN2 [56]. Except for birds and some other animals, all vertebrates have a paralogous *ATXN2L* gene, indicating that vertebrates originally had two *Ataxin-2* copies. This is most likely due to whole-genome duplication (WGD) events in early vertebrates prior to fish-tetrapod cleavage [57]. Other WGDs have been described for many lineages, including the very diverse infraclass *teleostei*, which accounts for about half of living vertebrates and over 96% of all fish species [58,59]. About 320 million years ago, the entire genome of teleost fishes underwent duplication, resulting in each gene in the genome receiving an additional copy. However, not all gene duplicates were retained [60,61,62,63]. Many ohnologous genes were probably redundant, and one copy was randomly lost in most species during evolution [62,64], which was probably also the case for *atxn2* and *atxn2l*, as both genes have only one copy in the zebrafish genome. This is an important aspect for the suitability of zebrafish as a model for ataxin-2 and ataxin-2l studies, as no functional redundancy due to ohnologous genes is to be expected. The effects of genetic alterations in *atxn2* and *atxn2l* are directly comparable to those in mammals.

We analyzed the phylogenetic relationship of Atxn2 and Atxn2l of zebrafish to proteins of other vertebrate models and used the human proteins as a reference. Two different clusters were found for ATXN2 and ATXN2L. The cladogram confirmed an identical relationship of ATXN2 between humans and chimpanzees (hominids) and a close relationship to ATXN2L. Other node cluster relationships are clearly shown for rodents (rat, mouse) or teleosts (zebrafish, killifish).

70% of human genes have at least one obvious ortholog in zebrafish, and 47% of human genes have a one-to-one relationship with an ortholog in zebrafish [65]. Since synteny refers to the presence of at least two or more genetic loci on chromosomes between species [66,67], no synteny could be found in zebrafish for the human *ATXN2* and its neighboring *SH2B3* gene (SH2B adapter protein 3), which belongs to a family of adapter proteins and influences a variety of signaling pathways [68]. Both ATXN2 and SH2B3, show an increased risk of ALS in the Turkish population when mutated [13]. Interestingly, the *SH2B1* gene, another member of the *SH2B* family, is located adjacent to *ATXN2L*. Both loci show synteny in the zebrafish genome. It remains unclear whether mutations in *ATXN2L* and *SH2B1* can also contribute to the risk of ALS. Here, the zebrafish could serve as an animal model for future studies.

Next, we focused on potential functional domains in the Atxn2 and Atxn2l proteins of zebrafish. The Ensembl database analysis showed two domains at the N-terminal end (SM-ATX, LsmAD) and a third PAM2 motif that are consistent with the known representative domains for ATXN2 and ATXNL in humans and other species [69,70,71,72,73,74]. The two known SM-ATX and LmsAD domains [75] interact with the DEAD-box helicase 6 (DDX6). The PABP-interacting motif PAM2 has been identified as an important binding site (pfam00658) in various eukaryotic proteins and found in a variety of eukaryotic proteins [56,71,76,77]. This motif interacts with PABP’s C-terminal MLLE domain. PABP is an RBP with function for polyA tailing, translation, mRNA decay and miRNA silencing [2,71,78].

We also used the Conserved Domain Algorithm (CD) search at NCBI for further domain analysis. The entries in the CDSEARCH/oasis_pfam database also showed the three matching protein domains (SM-ATX, LsmAD and PAM2) in zebrafish and human ATXN2 and ATXN2L. Interestingly, CDSEARCH/oasis_pfam analysis under concise and stringent search conditions identified another fourth domain in the C-terminal region of the zebrafish Atxn2, the human ATXN2 and ATXN2L, but not in the zebrafish Atxn2l. This fourth domain (pfam09770) has homology to the PAT1 protein family and was originally identified as a topoisomerase II-associated protein required for faithful chromosomal transcription in *Saccharomyces cerevisiae* [79]. The domain is conserved in eukaryotes and two PAT1 proteins (PATL1, PATL2) have been discovered in humans [80,81]. The proteins are involved in processing bodies formation [80,82], act as scaffolds that connect the deadenylation and decaffeination complexes in cytoplasmic mRNA turnover and regulate the biogenesis of hERG K^+^ channels via transcriptional mechanisms [83]. PATL1 can enter the cell nucleus, where it contributes to maintaining chromatin integrity and regulating transcription initiation [84]. It localizes to splicing speckles and is involved in RNA processes in both the nucleus and cytoplasm [85]. PAT1 is described as a hub for mRNA metabolism, acts in pre-mRNA splicing, translational repression, and mRNA decay [86,87]. Against this background, colocalization of ATXN2L and nuclear splice speckles have been observed, suggesting that ATXN2L may also play a role in splicing processes [76]. However, in contrast to this report, which describes a lack of the PAT1 domain in the human ATXN2 protein, our CDSEARCH/oasis_pfam results suggest a PAT1 domain for both human ATXN2 and ATXN2L proteins and zebrafish ATXN2. The PAT1 domain is not found in the zebrafish Atxn2l. As a summary result of our bioinformatic investigations, the zebrafish proteins Atxn2 and Atxn2l show a phylogenetic relationship to the human orthologues ATXN2 and ATXN2L, not only by the general sequence similarity, but also by domain characteristics of the proteins.

Despite knowledge of the spatial expression pattern of ATXN2 and ATXN2L in humans and model organisms, no expression patterns for *atxn2* and *atxn2l* have been described in zebrafish (ZFIN: https://zfin.org/), with the exception of a more recent detection in the trunk [53] and retina [47]. Here we provide initial data on the spatial and temporal expressions of the genes *atxn2* and *atxn2l* during early embryonic and larval development in zebrafish, which are described in detail in the ‘Results’ section of this study. Using our previously published method [52], we were able to detect the expression of *atxn2* already in the 1-cell stage of the zebrafish, suggesting a maternal contribution of *atxn2* transcripts, as well as in all stages of embryonic and larval development. *Atxn2* transcripts are detected in high concentrations in the developing brain. Tissue sections identified the telencephalon, the optic tectum, and the cerebellum as distinct expression domains of *atxn2* in zebrafish, as found in other vertebrates. ATXN2 and ATXN2L are both expressed in a wide range and are also found in high concentrations in the brain and non-neuronal tissues [72,88].

Our data show that the expression domains of *atxn2l* are largely the same as those of *atxn2* and are also detected in the zygotes, indicating a strong contribution of maternal *atxn2l* mRNA to the oocyte. In tissue sections, the telencephalon, the optic tectum and the cerebellum were identified as expression domains of *atxn2l.* Overall, the expression patterns of *atxn2* and *atxn2l* overlap. It remains unclear whether the expression of both genes is restricted to the same neuronal cells or to non-neuronal cells (glia) in different brain regions, and whether *atxn2* and *atxn2l* exhibit functional redundancy in zebrafish, which needs to be investigated in the future.

In addition to the annotated *atxn2* and the *atxn2l* transcripts in the Ensembl database, a BLAST search in the NCBI database provided many predicted isoforms for *atxn2* (X1–X28) and some for *atxn2l* (X1–X4) in zebrafish. The results showed amplified RT-PCR products spanning the entire annotated transcripts, including the extended predicted ends of the first and last exons in *atxn2* and the first exon of *atxn2l*. The data also showed developmentally regulated expression of *atxn2* during developmental stages up to 1 dpf, which is not the case for *atxn2l*, as a constant level of expression was observed at all developmental stages. However, both *atxn2* and *atxn2l* exhibit different splicing variants in the selected transcripts during development, which are not detectable in the brains of adult zebrafish. This implies that different isoforms of *atxn2* and *atxn2l* are required during zebrafish development, which are very likely to be expressed differently depending on the functional roles in different tissues and organs. Like ATXN2, ATXN2L is known as a direct RNA-binding protein and shares sequence homology with nuclear spliceosome factors [75,89] and alternatively spliced isoforms may interact with tyrosine kinase receptor signaling at the plasma membrane [55]. Isoforms of ATXN2 and ATXN2L, encoding a proline-rich domain, have been shown to mediate their direct association with SH3 motifs in components of the growth factor receptor endocytosis apparatus [73,90,91]. Abnormal splicing and alternative polyadenylation are also associated with ALS and related to RNA toxicity [6,92]. Reduced expression of ATXN2 may also contribute to the pathogenesis of ALS. Mutated TDP-43 protein aggregates are found in the brain and spinal cord of ALS patients. Therapeutic administration of antisense oligonucleotides for *Atxn2*, or crossing with *Atxn2*-deficient knockout mice has been shown to reduce TDP-43 aggregation, improve motor function, and extend lifespan in animal models [10]. Complete depletion of ATXN2 in knockout mice showed slight deficits in motor performance [38], reduced fertility, locomotor hyperactivity, abdominal obesity and hepatic steatosis in older animals [93], as well as effects on the circadian system [40].

Despite the spatial overlap of the *atxn2* and *atxn2l* gene expression domains, only *atxn2* expression is developmentally regulated. The transcripts of the *atxn2l* gene maintain steady levels and show different splicing patterns. It remains unclear whether both genes are co-expressed in the same cells in zebrafish and either have similar functions or not. Recent findings also point to non-neuronal functions. ATXN2L has been shown to be widely expressed in immortalized cell lines and in CD4-positive T-cell lymphomas [94]. ATXN2L may be involved in stress-related malignant activities of cancer, promoting cell invasiveness and can also be an indicator of gastric cancer [95]. ATXN2L contributes to diabetic peripheral neuropathy [96], the onset of type 2 diabetes in young people [97] and may be a promising therapeutic target for diabetic retinopathy [98]. CRISPR/Cas9-mediated deletion of Atxn2l exons 5–8 resulted in prenatal lethality of mouse fetuses with signs of lamination defects and apoptosis in neurons [6]. Taken together, this suggests a similar role of *atxn2l* in zebrafish as well, highlighting its special role as a potential model organism for non-neuronal and neurodegenerative diseases such as ALS and SCA2 and as a neuroprotective therapy model.

## 5. Conclusions

Our study attempts to bridge the gap in knowledge about *atxn2* and *atxn2l* genes in zebrafish which might be useful for future functional studies. Firstly, we have demonstrated that the absence of ohnologues makes disease modeling in zebrafish directly comparable to humans, particularly because we have demonstrated significant evolutionary conservation between the ATXN2 and ATXN2L proteins of humans and zebrafish. Secondly, we unraveled the spatio-temporal expression pattern of *atxn2* and *atxn2l* genes during zebrafish development, which is a prerequisite for both functional analysis and disease modeling studies. Thirdly, we suggest the zebrafish as a suitable animal model for functional studies and research in Atxn2- and Atxn2l-related diseases such al SCA2 and ALS. In addition to the well-known advantages of the zebrafish model, such as transparency and microscopability, low maintenance costs and genetic similarity to humans, its simple genetic modelability is particularly useful for biomedical studies. Genetic modeling could manifest SCA2 symptoms in zebrafish and would relate to the neurons that are targeted. Overexpression of pathogenic human ATXN2 in cerebellar Purkinje cells would be expected to lead to progressive degeneration of these neurons causing compromised locomotive behavior such as speed of swimming and turning behavior as well as an increase in anxiety (novel tank test, thigmotaxis). When motor neurons are targeted instead, again locomotive behavior such as distance and speed of swimming is expected to occur as phenotype but without altered anxiety behavior [99,100,101,102,103]. Alternatively, targeted replacement of zebrafish *atxn2* gene with a human polyQ-coding allele of *ATXN2,* or insertion of a polyQ-coding stretch into the zebrafish *atxn2* gene by the CRISPR/Cas9-mediated homologous recombination could be attempted. Based on the data presented in this study, we strongly recommend zebrafish as a suitable model for future experiments modeling neurodegenerative diseases.

## Data Availability

The authors state that all data (sequences) used in this study are publicly available in the NCBI and Ensembl databases, as specified in the article. The original contributions presented in this study are included in the article and Supplementary Materials. Further inquiries can be directed to the corresponding author.

## Supplementary Materials

The following supporting information can be downloaded at: https://www.mdpi.com/article/10.3390/biomedicines13122974/s1, Table S1: Ataxin 2 and Ataxin 2-like proteins in vertebrates; Table S2: Selected upstream (U) and lower (L) primer positions in corresponding cDNA for RT-PCR and probe synthesis.

## Abbreviations

The following abbreviations are used in this manuscript:

ATXN2	Ataxin-2
ATXN2L	Ataxin-2-like
ALS	Amyotrophic lateral sclerosis
FTLD	Frontotemporal lobar dementia

## References

[1] Choudhry S. (2001). CAG Repeat Instability at SCA2 Locus: Anchoring CAA Interruptions and Linked Single Nucleotide Polymorphisms. Hum. Mol. Genet..

[2] Kozlov G., Trempe J.-F., Khaleghpour K., Kahvejian A., Ekiel I., Gehring K. (2001). Structure and Function of the C-Terminal PABC Domain of Human Poly(A)-Binding Protein. Proc. Natl. Acad. Sci. USA.

[3] Yokoshi M., Li Q., Yamamoto M., Okada H., Suzuki Y., Kawahara Y. (2014). Direct Binding of Ataxin-2 to Distinct Elements in 3′ UTRs Promotes mRNA Stability and Protein Expression. Mol. Cell.

[4] Auburger G., Sen N.-E., Meierhofer D., Başak A.-N., Gitler A.D. (2017). Efficient Prevention of Neurodegenerative Diseases by Depletion of Starvation Response Factor Ataxin-2. Trends Neurosci..

[5] Van De Loo S., Eich F., Nonis D., Auburger G., Nowock J. (2009). Ataxin-2 Associates with Rough Endoplasmic Reticulum. Exp. Neurol..

[6] Key J., Harter P.N., Sen N.-E., Gradhand E., Auburger G., Gispert S. (2020). Mid-Gestation Lethality of Atxn2l-Ablated Mice. Int. J. Mol. Sci..

[7] Imbert G., Saudou F., Yvert G., Devys D., Trottier Y., Garnier J.-M., Weber C., Mandel J.-L., Cancel G., Abbas N. (1996). Cloning of the Gene for Spinocerebellar Ataxia 2 Reveals a Locus with High Sensitivity to Expanded CAG/Glutamine Repeats. Nat. Genet..

[8] Pulst S.-M., Nechiporuk A., Nechiporuk T., Gispert S., Chen X.-N., Lopes-Cendes I., Pearlman S., Starkman S., Orozco-Diaz G., Lunkes A. (1996). Moderate Expansion of a Normally Biallelic Trinucleotide Repeat in Spinocerebellar Ataxia Type 2. Nat. Genet..

[9] Sanpei K., Takano H., Igarashi S., Sato T., Oyake M., Sasaki H., Wakisaka A., Tashiro K., Ishida Y., Ikeuchi T. (1996). Identification of the Spinocerebellar Ataxia Type 2 Gene Using a Direct Identification of Repeat Expansion and Cloning Technique, DIRECT. Nat. Genet..

[10] Becker L.A., Huang B., Bieri G., Ma R., Knowles D.A., Jafar-Nejad P., Messing J., Kim H.J., Soriano A., Auburger G. (2017). Therapeutic Reduction of Ataxin-2 Extends Lifespan and Reduces Pathology in TDP-43 Mice. Nature.

[11] Elden A.C., Kim H.-J., Hart M.P., Chen-Plotkin A.S., Johnson B.S., Fang X., Armakola M., Geser F., Greene R., Lu M.M. (2010). Ataxin-2 Intermediate-Length Polyglutamine Expansions Are Associated with Increased Risk for ALS. Nature.

[12] Gispert S., Kurz A., Waibel S., Bauer P., Liepelt I., Geisen C., Gitler A.D., Becker T., Weber M., Berg D. (2012). The Modulation of Amyotrophic Lateral Sclerosis Risk by Ataxin-2 Intermediate Polyglutamine Expansions Is a Specific Effect. Neurobiol. Dis..

[13] Lahut S., Ömür Ö., Uyan Ö., Ağım Z.S., Özoğuz A., Parman Y., Deymeer F., Oflazer P., Koç F., Özçelik H. (2012). ATXN2 and Its Neighbouring Gene SH2B3 Are Associated with Increased ALS Risk in the Turkish Population. PLoS ONE.

[14] Lee T., Li Y.R., Ingre C., Weber M., Grehl T., Gredal O., de Carvalho M., Meyer T., Tysnes O.-B., Auburger G. (2011). Ataxin-2 Intermediate-Length Polyglutamine Expansions in European ALS Patients. Hum. Mol. Genet..

[15] Ross O.A., Rutherford N.J., Baker M., Soto-Ortolaza A.I., Carrasquillo M.M., DeJesus-Hernandez M., Adamson J., Li M., Volkening K., Finger E. (2011). Ataxin-2 Repeat-Length Variation and Neurodegeneration. Hum. Mol. Genet..

[16] Shulman J.M., Feany M.B. (2003). Genetic Modifiers of Tauopathy in Drosophila. Genetics.

[17] Andrés A.M., Lao O., Soldevila M., Calafell F., Bertranpetit J. (2003). Dynamics of CAG Repeat Loci Revealed by the Analysis of Their Variability: CAG REPEAT LOCI DYNAMICS. Hum. Mutat..

[18] Paulson H., Geschwind D.H., Paulson H.L., Klein C. (2018). Chapter 9-Repeat Expansion Diseases. Handbook of Clinical Neurology.

[19] Schöls L., Bauer P., Schmidt T., Schulte T., Riess O. (2004). Autosomal Dominant Cerebellar Ataxias: Clinical Features, Genetics, and Pathogenesis. Lancet Neurol..

[20] Taroni F., DiDonato S. (2004). Pathways to Motor Incoordination: The Inherited Ataxias. Nat. Rev. Neurosci..

[21] Costa R.G., Conceição A., Matos C.A., Nóbrega C. (2024). The Polyglutamine Protein ATXN2: From Its Molecular Functions to Its Involvement in Disease. Cell Death Dis..

[22] Hekman K.E., Gomez C.M. (2015). The Autosomal Dominant Spinocerebellar Ataxias: Emerging Mechanistic Themes Suggest Pervasive Purkinje Cell Vulnerability. J. Neurol. Neurosurg. Psychiatry.

[23] Orr H.T., Chung M., Banfi S., Kwiatkowski T.J., Servadio A., Beaudet A.L., McCall A.E., Duvick L.A., Ranum L.P.W., Zoghbi H.Y. (1993). Expansion of an Unstable Trinucleotide CAG Repeat in Spinocerebellar Ataxia Type 1. Nat. Genet..

[24] Perlman S. Hereditary Ataxia Overview. 1998 Oct 28 [Updated 2012 May 31]. GeneReviews^TM^Internet Seattle WA Univ. Wash. Seattle 1993. http://www.ncbi.nlm.nih.gov/books/NBK1138/.

[25] Housman D. (1995). Gain of Glutamines, Gain of Function?. Nat. Genet..

[26] Manto M., Marmolino D. (2009). Cerebellar Ataxias. Curr. Opin. Neurol..

[27] Quelle-Regaldie A., Sobrido-Cameán D., Barreiro-Iglesias A., Sobrido M.J., Sánchez L. (2021). Zebrafish Models of Autosomal Dominant Ataxias. Cells.

[28] Cancel G., Durr A., Didierjean O., Imbert G., Burk K., Lezin A., Belal S., Benomar A., Abada-Bendib M., Vial C. (1997). Molecular and Clinical Correlations in Spinocerebellar Ataxia 2: A Study of 32 Families. Hum. Mol. Genet..

[29] Geschwind D.H., Perlman S., Pulst S.M. (1997). The Prevalence and Wide Clinical Spectrum of the Spinocerebellar Ataxia Type 2 Trinucleotide Repeat in Patients with Autosomal Dominant Cerebellar Ataxia. Am. J. Hum. Genet..

[30] Giunti P. (1998). The Role of the SCA2 Trinucleotide Repeat Expansion in 89 Autosomal Dominant Cerebellar Ataxia Families. Frequency, Clinical and Genetic Correlates. Brain.

[31] Mizushima K., Watanabe M., Abe K., Aoki M., Itoyama Y., Shizuka M., Okamoto K., Shoji M. (1998). Analysis of Spinocerebellar Ataxia Type 2 in Gunma Prefecture in Japan: CAG Trinucleotide Expansion and Clinical Characteristics. J. Neurol. Sci..

[32] Auburger G., Diaz G.O., Perez M.P., Williamson R., Chamberlain S., Bautet L.H. (1990). Autosomal Dominant Ataxia: Genetic Evidence for Locus Heterogeneity from a Cuban Founder-Effect Population. Am. J. Hum. Genet..

[33] Tsuji S., Onodera O., Goto J., Nishizawa M., On Behalf of the Study Group on Ataxic Diseases (2008). Sporadic Ataxias in Japan—A Population-Based Epidemiological Study. Cerebellum.

[34] Cruz-Mariño T., Vázquez-Mojena Y., Velázquez-Pérez L., González-Zaldívar Y., Aguilera-Rodríguez R., Velázquez-Santos M., Estupiñán-Rodríguez A., Laffita-Mesa J.M., Almaguer-Mederos L.E., Paneque M. (2015). SCA2 Predictive Testing in Cuba: Challenging Concepts and Protocol Evolution. J. Community Genet..

[35] Estrada R., Galarraga J., Orozco G., Nodarse A., Auburger G. (1999). Spinocerebellar Ataxia 2 (SCA2): Morphometric Analyses in 11 Autopsies. Acta Neuropathol..

[36] Velázquez Pérez L., Cruz G.S., Santos Falcón N., Enrique Almaguer Mederos L., Escalona Batallan K., Rodríguez Labrada R., Paneque Herrera M., Laffita Mesa J.M., Rodríguez Díaz J.C., Rodríguez R.A. (2009). Molecular Epidemiology of Spinocerebellar Ataxias in Cuba: Insights into SCA2 Founder Effect in Holguin. Neurosci. Lett..

[37] Velázquez-Pérez L.C., Rodríguez-Labrada R., Fernandez-Ruiz J. (2017). Spinocerebellar Ataxia Type 2: Clinicogenetic Aspects, Mechanistic Insights, and Management Approaches. Front. Neurol..

[38] Kiehl T.-R., Nechiporuk A., Figueroa K.P., Keating M.T., Huynh D.P., Pulst S.-M. (2006). Generation and Characterization of Sca2 (Ataxin-2) Knockout Mice. Biochem. Biophys. Res. Commun..

[39] Lastres-Becker I., Nonis D., Eich F., Klinkenberg M., Gorospe M., Kötter P., Klein F.A.C., Kedersha N., Auburger G. (2016). Mammalian Ataxin-2 Modulates Translation Control at the Pre-Initiation Complex via PI3K/mTOR and Is Induced by Starvation. Biochim. Biophys. Acta BBA Mol. Basis Dis..

[40] Pfeffer M., Gispert S., Auburger G., Wicht H., Korf H.-W. (2017). Impact of Ataxin-2 Knock out on Circadian Locomotor Behavior and PER Immunoreaction in the SCN of Mice. Chronobiol. Int..

[41] Lim C., Allada R. (2013). ATAXIN-2 Activates PERIOD Translation to Sustain Circadian Rhythms in *Drosophila*. Science.

[42] Zhang Y., Ling J., Yuan C., Dubruille R., Emery P. (2013). A Role for *Drosophila* ATX2 in Activation of PER Translation and Circadian Behavior. Science.

[43] Vieira De Sá R., Sudria-Lopez E., Cañizares Luna M., Harschnitz O., Van Den Heuvel D.M.A., Kling S., Vonk D., Westeneng H.-J., Karst H., Bloemenkamp L. (2024). ATAXIN-2 Intermediate-Length Polyglutamine Expansions Elicit ALS-Associated Metabolic and Immune Phenotypes. Nat. Commun..

[44] Van Den Heuvel D.M.A., Harschnitz O., Van Den Berg L.H., Pasterkamp R.J. (2014). Taking a Risk: A Therapeutic Focus on Ataxin-2 in Amyotrophic Lateral Sclerosis?. Trends Mol. Med..

[45] Ciura S., Sellier C., Campanari M.-L., Charlet-Berguerand N., Kabashi E. (2016). The Most Prevalent Genetic Cause of ALS-FTD, C9orf72 Synergizes the Toxicity of ATXN2 Intermediate Polyglutamine Repeats through the Autophagy Pathway. Autophagy.

[46] Sellier C., Campanari M., Julie Corbier C., Gaucherot A., Kolb-Cheynel I., Oulad-Abdelghani M., Ruffenach F., Page A., Ciura S., Kabashi E. (2016). Loss of C9 ORF 72 Impairs Autophagy and Synergizes with polyQ Ataxin-2 to Induce Motor Neuron Dysfunction and Cell Death. EMBO J..

[47] Song Rong S., Larson A., Wiggs J.L. (2025). ATXN2 Loss of Function Results in Glaucoma-Related Features Supporting a Role for Ataxin-2 in Primary Open-Angle Glaucoma (POAG) Pathogenesis. Vision Res..

[48] Okonechnikov K., Golosova O., Fursov M., The UGENE Team (2012). Unipro UGENE: A Unified Bioinformatics Toolkit. Bioinformatics.

[49] Wang J., Chitsaz F., Derbyshire M.K., Gonzales N.R., Gwadz M., Lu S., Marchler G.H., Song J.S., Thanki N., Yamashita R.A. (2023). The Conserved Domain Database in 2023. Nucleic Acids Res..

[50] Blum M., Andreeva A., Florentino L.C., Chuguransky S.R., Grego T., Hobbs E., Pinto B.L., Orr A., Paysan-Lafosse T., Ponamareva I. (2025). InterPro: The Protein Sequence Classification Resource in 2025. Nucleic Acids Res..

[51] Paysan-Lafosse T., Andreeva A., Blum M., Chuguransky S.R., Grego T., Pinto B.L., Salazar G.A., Bileschi M.L., Llinares-López F., Meng-Papaxanthos L. (2025). The Pfam Protein Families Database: Embracing AI/ML. Nucleic Acids Res..

[52] Vauti F., Stegemann L.A., Vögele V., Köster R.W. (2020). All-Age Whole Mount in Situ Hybridization to Reveal Larval and Juvenile Expression Patterns in Zebrafish. PLoS ONE.

[53] Laboissonniere L.A., Smith C.L., Mesenbrink J., Chowdhury R., Burney A., Lang M., Sierra M., Stark A., Maldonado-Casalduc G., Muller M. (2018). ALS-Associated Genes Display CNS Expression in the Developing Zebrafish. Gene Expr. Patterns.

[54] Laffita-Mesa J.M., Paucar M., Svenningsson P. (2021). Ataxin-2 Gene: A Powerful Modulator of Neurological Disorders. Curr. Opin. Neurol..

[55] Meunier C., Bordereaux D., Porteu F., Gisselbrecht S., Chrétien S., Courtois G. (2002). Cloning and Characterization of a Family of Proteins Associated with Mpl. J. Biol. Chem..

[56] Jiménez-López D., Guzmán P. (2014). Insights into the Evolution and Domain Structure of Ataxin-2 Proteins across Eukaryotes. BMC Res. Notes.

[57] Dehal P., Boore J.L. (2005). Two Rounds of Whole Genome Duplication in the Ancestral Vertebrate. PLoS Biol..

[58] Faircloth B.C., Sorenson L., Santini F., Alfaro M.E. (2013). A Phylogenomic Perspective on the Radiation of Ray-Finned Fishes Based upon Targeted Sequencing of Ultraconserved Elements (UCEs). PLoS ONE.

[59] Sallan L.C. (2014). Major Issues in the Origins of Ray-finned Fish (A Ctinopterygii) Biodiversity. Biol. Rev..

[60] Hoegg S., Brinkmann H., Taylor J.S., Meyer A. (2004). Phylogenetic Timing of the Fish-Specific Genome Duplication Correlates with the Diversification of Teleost Fish. J. Mol. Evol..

[61] Jaillon O., Aury J.-M., Brunet F., Petit J.-L., Stange-Thomann N., Mauceli E., Bouneau L., Fischer C., Ozouf-Costaz C., Bernot A. (2004). Genome Duplication in the Teleost Fish Tetraodon Nigroviridis Reveals the Early Vertebrate Proto-Karyotype. Nature.

[62] Sémon M., Wolfe K.H. (2007). Reciprocal Gene Loss between Tetraodon and Zebrafish after Whole Genome Duplication in Their Ancestor. Trends Genet..

[63] Van de Peer Y., Taylor J.S., Meyer A., Meyer A., Van de Peer Y. (2003). Are All Fishes Ancient Polyploids?. Genome Evolution.

[64] Semon M., Wolfe K.H. (2006). Rearrangement Rate Following the Whole-Genome Duplication in Teleosts. Mol. Biol. Evol..

[65] Howe K., Clark M.D., Torroja C.F., Torrance J., Berthelot C., Muffato M., Collins J.E., Humphray S., McLaren K., Matthews L. (2013). The Zebrafish Reference Genome Sequence and Its Relationship to the Human Genome. Nature.

[66] Renwick J.H. (1971). The Mapping of Human Chromosomes. Annu. Rev. Genet..

[67] Veltri D., Wight M.M., Crouch J.A. (2016). SimpleSynteny: A Web-Based Tool for Visualization of Microsynteny across Multiple Species. Nucleic Acids Res..

[68] Maures T.J., Kurzer J.H., Carter-Su C. (2007). SH2B1 (SH2-B) and JAK2: A Multifunctional Adaptor Protein and Kinase Made for Each Other. Trends Endocrinol. Metab..

[69] Albrecht M., Golatta M., Wüllner U., Lengauer T. (2004). Structural and Functional Analysis of Ataxin-2 and Ataxin-3. Eur. J. Biochem..

[70] Huynh D.P., Figueroa K., Hoang N., Pulst S.-M. (2000). Nuclear Localization or Inclusion Body Formation of Ataxin-2 Are Not Necessary for SCA2 Pathogenesis in Mouse or Human. Nat. Genet..

[71] Lee J., Kim M., Itoh T.Q., Lim C. (2018). Ataxin-2: A Versatile Posttranscriptional Regulator and Its Implication in Neural Function. WIREs RNA.

[72] Li L., Wang M., Huang L., Zheng X., Wang L., Miao H. (2024). Ataxin-2: A Powerful RNA-Binding Protein. Discov. Oncol..

[73] Nonis D., Schmidt M.H.H., van de Loo S., Eich F., Dikic I., Nowock J., Auburger G. (2008). Ataxin-2 Associates with the Endocytosis Complex and Affects EGF Receptor Trafficking. Cell. Signal..

[74] Satterfield T.F., Pallanck L.J. (2006). Ataxin-2 and Its Drosophila Homolog, ATX2, Physically Assemble with Polyribosomes. Hum. Mol. Genet..

[75] Figueroa K.P., Pulst S.M. (2003). Identification and Expression of the Gene for Human Ataxin-2-Related Protein on Chromosome 16. Exp. Neurol..

[76] Kaehler C., Isensee J., Nonhoff U., Terrey M., Hucho T., Lehrach H., Krobitsch S. (2012). Ataxin-2-Like Is a Regulator of Stress Granules and Processing Bodies. PLoS ONE.

[77] Tharun S. (2008). Chapter 4 Roles of Eukaryotic Lsm Proteins in the Regulation of mRNA Function. International Review of Cell and Molecular Biology.

[78] Kozlov G., Ménade M., Rosenauer A., Nguyen L., Gehring K. (2010). Molecular Determinants of PAM2 Recognition by the MLLE Domain of Poly(A)-Binding Protein. J. Mol. Biol..

[79] Wang X. (1996). Pat1: A Topoisomerase II-Associated Protein Required for Faithful Chromosome Transmission in Saccharomyces Cerevisiae. Nucleic Acids Res..

[80] Ozgur S., Chekulaeva M., Stoecklin G. (2010). Human Pat1b Connects Deadenylation with mRNA Decapping and Controls the Assembly of Processing Bodies. Mol. Cell. Biol..

[81] Scheller N., Resa-Infante P., de la Luna S., Galao R.P., Albrecht M., Kaestner L., Lipp P., Lengauer T., Meyerhans A., Díez J. (2007). Identification of PatL1, a Human Homolog to Yeast P Body Component Pat1. Biochim. Biophys. Acta BBA-Mol. Cell Res..

[82] Braun J.E., Tritschler F., Haas G., Igreja C., Truffault V., Weichenrieder O., Izaurralde E. (2010). The C-Terminal α–α Superhelix of Pat Is Required for mRNA Decapping in Metazoa. EMBO J..

[83] Yao L., Ruan M.-Y., Ye S.-W., Cai S.-Q. (2023). DNA Topoisomerase 2-Associated Proteins PATL1 and PATL2 Regulate the Biogenesis of hERG K^+^ Channels. Proc. Natl. Acad. Sci. USA.

[84] Standart N., Marnef A. (2012). Pat1 Proteins: Regulating mRNAs from Birth to Death?. Biomol. Concepts.

[85] Marnef A., Weil D., Standart N. (2012). RNA-Related Nuclear Functions of Human Pat1b, the P-Body mRNA Decay Factor. Mol. Biol. Cell.

[86] He F., Celik A., Wu C., Jacobson A. (2018). General Decapping Activators Target Different Subsets of Inefficiently Translated mRNAs. eLife.

[87] Lobel J.H., Tibble R.W., Gross J.D. (2019). Pat1 Activates Late Steps in mRNA Decay by Multiple Mechanisms. Proc. Natl. Acad. Sci. USA.

[88] Ostrowski L., Hall A., Mekhail K. (2017). Ataxin-2: From RNA Control to Human Health and Disease. Genes.

[89] Neuwald A.F., Koonin E.V. (1998). Ataxin-2, Global Regulators of Bacterial Gene Expression, and Spliceosomal snRNP Proteins Share a Conserved Domain. J. Mol. Med. Berl. Ger..

[90] Drost J., Nonis D., Eich F., Leske O., Damrath E., Brunt E.R., Lastres-Becker I., Heumann R., Nowock J., Auburger G. (2013). Ataxin-2 Modulates the Levels of Grb2 and Src but Not Ras Signaling. J. Mol. Neurosci..

[91] Lastres-Becker I., Nonis D., Nowock J., Auburger G. (2019). New Alternative Splicing Variants of the ATXN2 Transcript. Neurol. Res. Pract..

[92] Prudencio M., Belzil V.V., Batra R., Ross C.A., Gendron T.F., Pregent L.J., Murray M.E., Overstreet K.K., Piazza-Johnston A.E., Desaro P. (2015). Distinct Brain Transcriptome Profiles in C9orf72-Associated and Sporadic ALS. Nat. Neurosci..

[93] Lastres-Becker I., Brodesser S., Lütjohann D., Azizov M., Buchmann J., Hintermann E., Sandhoff K., Schürmann A., Nowock J., Auburger G. (2008). Insulin Receptor and Lipid Metabolism Pathology in Ataxin-2 Knock-out Mice. Hum. Mol. Genet..

[94] Panagopoulos I., Gorunova L., Spetalen S., Bassarova A., Beiske K., Micci F., Heim S. (2017). Fusion of the Genes Ataxin 2 like, *ATXN2L*, and Janus Kinase 2, *JAK2*, in Cutaneous CD4 Positive T-Cell Lymphoma. Oncotarget.

[95] Lin L., Li X., Pan C., Lin W., Shao R., Liu Y., Zhang J., Luo Y., Qian K., Shi M. (2019). ATXN2L Upregulated by Epidermal Growth Factor Promotes Gastric Cancer Cell Invasiveness and Oxaliplatin Resistance. Cell Death Dis..

[96] Gong X., Gui Z., Ye X., Li X. (2023). Jatrorrhizine Ameliorates Schwann Cell Myelination via Inhibiting HDAC3 Ability to Recruit Atxn2l for Regulating the NRG1-ERBB2-PI3K-AKT Pathway in Diabetic Peripheral Neuropathy Mice. Phytother. Res..

[97] Kwak S.H., Srinivasan S., Chen L., Todd J., Mercader J.M., Jensen E.T., Divers J., Mottl A.K., Pihoker C., Gandica R.G. (2024). Genetic Architecture and Biology of Youth-Onset Type 2 Diabetes. Nat. Metab..

[98] Niu C., Dong D., Cui L., Dong Y., Wang W. (2025). Exosomal FOXL1 from Bone Marrow Mesenchymal Stem Cells Activates the METTL3/ATXN2L Pathway to Ameliorate High Glucose-Induced Human Retinal Microvascular Endothelial Cell Injury. Diabetol. Metab. Syndr..

[99] Watchon M., Yuan K.C., Mackovski N., Svahn A.J., Cole N.J., Goldsbury C., Rinkwitz S., Becker T.S., Nicholson G.A., Laird A.S. (2017). Calpain Inhibition Is Protective in Machado–Joseph Disease Zebrafish Due to Induction of Autophagy. J. Neurosci..

[100] Elsaey M.A., Namikawa K., Köster R.W. (2021). Genetic Modeling of the Neurodegenerative Disease Spinocerebellar Ataxia Type 1 in Zebrafish. Int. J. Mol. Sci..

[101] Namikawa K., Dorigo A., Zagrebelsky M., Russo G., Kirmann T., Fahr W., Dübel S., Korte M., Köster R.W. (2019). Modeling Neurodegenerative Spinocerebellar Ataxia Type 13 in Zebrafish Using a Purkinje Neuron Specific Tunable Coexpression System. J. Neurosci..

[102] Namikawa K., Dorigo A., Köster R.W. (2019). Neurological Disease Modelling for Spinocerebellar Ataxia Using Zebrafish. J. Exp. Neurosci..

[103] Buchberger A., Schepergerdes L., Flaßhoff M., Kunick C., Köster R.W. (2021). A Novel Inhibitor Rescues Cerebellar Defects in a Zebrafish Model of Down Syndrome–Associated Kinase Dyrk1A Overexpression. J. Biol. Chem..

[104] Aleström P., D’Angelo L., Midtlyng P.J., Schorderet D.F., Schulte-Merker S., Sohm F., Warner S. (2020). Zebrafish: Housing and Husbandry Recommendations. Lab. Anim..

