# Supplementary material for: Genomic Organization, Evolutionary Conservation and Expression of Ataxin-2 and Ataxin-2-like Genes Underscore the Suitability of Zebrafish as a Model Organism for SCA2 and Related Diseases
Source: Biomedicines. 2025 Dec 3;13(12):2974. doi: 10.3390/biomedicines13122974 (PMC12730607; doi:10.3390/biomedicines13122974)
Supplement: Supplementary file 1 [file biomedicines-13-02974-s001.zip › Supplementary Table S2.pdf]

Table S2: Selected upstream (U) and lower (L) primer positions in corresponding cDNA for RT-PCR and probe synthesis

Primer Position	***atxn2*** **Transcript**	Sequence	Fragment length	Remark
**U-57**	57 nt extended 5´-UTR (by DNA-ID: CR848794.6)	CCACTTTTTGTGGCTTTTCAGACTGTTTAGG	**550 bp**	primer binds to extended 5´-UTR (from DNA sequence)
**L493**	atxn 2 Transcript ID: ENSDART00000083656.5	GTCCTTTGGCATTGTTGCGTCCTC		
**U-57**	57 nt extended 5´-UTR (by DNA-ID: CR848794.6)	CCACTTTTTGTGGCTTTTCAGACTGTTTAGG	**1460 bp**	primer binds to extended 5´-UTR (from DNA sequence)
**L1403**	atxn 2 Transcript ID: ENSDART00000083656.5	CGAGAGGAGGGAGATGGGCAG		
**U381**	atxn 2 Transcript ID: ENSDART00000083656.5	TCGCAAACCCGGCGGAAG	**1971 bp**	
**L2351**	atxn 2 Transcript ID: ENSDART00000083656.5	GGGACAGCAGAGGAAGGAGAGAG		
**U1384**	atxn 2 Transcript ID: ENSDART00000083656.5	TGCCCATCTCCCTCCTCTCG	**1952 bp**	
**L3335**	atxn 2 Transcript ID: ENSDART00000083656.5	TGCTGCTGCTGTGGGTTGC		
**U2322**	atxn 2 Transcript ID: ENSDART00000083656.5	CCCTTCGCTCTCTCCTTCCTCTG	**1337 bp**	
**L3658**	atxn 2 Transcript ID: ENSDART00000083656.5	GGGTGAGTGTGGAGTAGAAGTGTGTG		
**U3162**	atxn 2 Transcript ID: ENSDART00000083656.5	GCACCCTCATCCACAGCACC	**1169 bp**	
**(L+126) = L4330**	126 nt extended 3´-UTR by X1: XM_009301663.5	TTTATATTATCTACAAAGTAAAAGTTATTTTAACTTAACAGTTCTTATAACGGTCAAG		primer binds to extended 3´-UTR sequence (X1 variant)
**U3377**	atxn 2 Transcript ID: ENSDART00000083656.5	CCGGCCCAAACCCACAGTC	**954 bp**	
**(L+126) = L4330**	126 nt extended 3´-UTR by X1: XM_009301663.5	TTTATATTATCTACAAAGTAAAAGTTATTTTAACTTAACAGTTCTTATAACGGTCAAG		primer binds to extended 3´-UTR sequence (X1 variant)
**U2322**	atxn 2 Transcript ID: ENSDART00000083656.5	CCCTTCGCTCTCTCCTTCCTCTG	**1358 nt**	primer for *atxn2* probe (WISH)
**L3679**	atxn 2 Transcript ID: ENSDART00000083656.5	GCTGATGGTGAGCTTGCACAGG		

Primer Position	***atxn2l*** **Transcript**	Sequence	Fragment length	Remark
**U-27**	27 nt extended 5´- UTR (by DNA-ID: BX784026.17)	GTCGAAGGTTTTTGAAAAAACAGCCATCG	**637 bp**	primer binds to extended 5´-UTR (from DNA sequence)
**L610**	atxn 2-like Transcript ID: ENSDART00000133168.3	CCGCCTCCATCTCCTTCCTCATTC		
**U-27**	27 nt extended 5´- UTR (by DNA-ID: BX784026.17)	GTCGAAGGTTTTTGAAAAAACAGCCATCG	**1444 bp**	primer binds to extended 5´-UTR (from DNA sequence)
**L1417**	atxn 2-like Transcript ID: ENSDART00000133168.3	GGTGTGCTGCTCTGTGATTGGTG		
**U1202**	atxn 2-like Transcript ID: ENSDART00000133168.3	GAGAGCAAGGGAGCGAGAGATGG	**2111 bp**	
**L3312**	atxn 2-like Transcript ID: ENSDART00000133168.3	GCGCTCTCTCTTTCTCTCCCTCTC		
**U2280**	atxn 2-like Transcript ID: ENSDART00000133168.3	CCCACACCACCACGACCAAC	**1402 bp**	
**L3681**	atxn 2-like Transcript ID: ENSDART00000133168.3	GAGAGTTTAAAAATTTATTTTTTATACATTTTTTTTTATTTTTTTTGTTCTTTTTTTAACCATTTATTATTTGC		
**U3023**	atxn 2-like Transcript ID: ENSDART00000133168.3	GCCGCATCCTTACTCCTCTCTGG	**659 bp**	
**L3681**	atxn 2-like Transcript ID: ENSDART00000133168.3	GAGAGTTTAAAAATTTATTTTTTATACATTTTTTTTTATTTTTTTTGTTCTTTTTTTAACCATTTATTATTTGC		
**U1106**	atxn 2-like Transcript ID: ENSDART00000133168.3	AGACCGGGAGCGTGAAGCA	**1192 bp**	primer for *atxn2l* probe (WISH)
**L2297**	atxn 2-like Transcript ID: ENSDART00000133168.3	TGGTCGTGGTGGTGTGGGA		

Primer Position	***β-actin*** **Transcript**	Sequence	Fragment length	Remark
**U67**	β-actin NM_131031	TCCCCTTGTTCACAATAACC	**383 bp**	primer for control (β-actin)
**L449**	β-actin NM_131031	TCTGTTGGCTTTGGGATTC		
