# Supplementary material for: Genomic Organization, Evolutionary Conservation and Expression of Ataxin-2 and Ataxin-2-like Genes Underscore the Suitability of Zebrafish as a Model Organism for SCA2 and Related Diseases
Source: Biomedicines. 2025 Dec 3;13(12):2974. doi: 10.3390/biomedicines13122974 (PMC12730607; doi:10.3390/biomedicines13122974)
Supplement: Supplementary file 1 [file biomedicines-13-02974-s001.zip › Supplementary Table S1.pdf]

| **Table S1**. Ataxin 2 and Ataxin 2-like proteins in vertebrates | | | |
|---|---|---|---|
| **Vertebrate species** | **Accession number** | **Protein** | **Amino acid residues** |
| *Homo sapiens* | Q99700.2 | Human ATXN2 | 1313 aa |
| *Pan troglodytes* | PNI64641.1 | Chimpanzee ATXN2 | 1315 aa |
| *Rattus norvegicus* | NP_001406666.1 | Rat ATXN2 | 1136 aa |
| *Mus musculus* | NP_033151.3 | Mouse ATXN2 | 1136 aa |
| *Gallus gallus* | XP_015131043.2 | Chicken ATXN2 | 1121 aa |
| *Xenopus tropicalis* | XP_002937742.3 | Frog Atxn2 | 1082 aa |
| *Danio rerio* | NP_001121821.1 | Zebrafish Atxn2 | 1112 aa |
| *Nothobranchius furzeri* | XP_015828412.2 | Killifish Atxn2 | 1121 aa |
| | | | |
| *Homo sapiens* | NP_009176.2 | Human ATXN2L | 1075 aa |
| *Pan troglodytes* | XP_016784194.1 | Chimpanzee ATXN2L | 1103 aa |
| *Rattus norvegicus* | NP_001123569.2 | Rat ATXN2L | 1072 aa |
| *Mus musculus* | NP_001348416.1 | Mouse ATXN2L | 1074 aa |
| *Xenopus tropicalis* | XP_031748418.1 | Frog Atxn2l | 971 aa |
| *Danio rerio* | NP_997849.3 | Zebrafish Atxn2l | 1004 aa |
| *Nothobranchius furzeri* | XP_070401698 | Killifish Atxn2l | 1017 aa |
